# Incidence and predictors of surgical recurrence after primary ileocolic resection in Crohn ileitis: a systematic review and meta-analysis

**DOI:** 10.1093/ibd/izag037

**Published:** 2026-04-19

**Authors:** Anouck E G Haanappel, Eline M L van der Does de Willebois, Mahsoem Ali, Faridi S Jamaludin, Marjolijn Duijvestein, Christianne J Buskens, Willem A Bemelman, E Joline de Groof

**Affiliations:** Amsterdam UMC, location Vrije Universiteit, Department of Surgery, Amsterdam, The Netherlands; Amsterdam UMC, location Vrije Universiteit, Department of Surgery, Amsterdam, The Netherlands; Amsterdam UMC, location Vrije Universiteit, Department of Surgery, Amsterdam, The Netherlands; Medical Library AMC, Amsterdam UMC, University of Amsterdam, Amsterdam, The Netherlands; Department of Gastroenterology, Radboud UMC, Nijmegen, The Netherlands; Amsterdam UMC, location Vrije Universiteit, Department of Surgery, Amsterdam, The Netherlands; Amsterdam UMC, location Vrije Universiteit, Department of Surgery, Amsterdam, The Netherlands; Amsterdam UMC, location Vrije Universiteit, Department of Surgery, Amsterdam, The Netherlands

**Keywords:** ileocecal resection, Crohn disease, surgical recurrence, biologics

## Abstract

**Introduction:**

Treatment strategies for Crohn disease (CD) have evolved considerably over recent decades. Approximately 65% of primary resections for CD comprise ileocolic resections (ICR). Currently, up-to-date data are not available on surgical recurrence risk after primary ICR, as previous meta-analyses combined various types of intestinal resections. As such, we aimed to assess the incidence, predictors, and trends of surgical recurrence after primary ICR for CD.

**Methods:**

MEDLINE, EMBASE, and the Cochrane database were searched from inception until November 15, 2023. Studies were included if they described the surgical recurrence risk after a primary ICR for CD. The primary endpoint was surgical recurrence, defined as reoperation due to disease recurrence in the neoterminal ileum. Meta-analyses were performed to (1) evaluate trends in surgical recurrence over time, (2) estimate the risk of surgical recurrence in the biologics era (post-2000), and (3) identify risk factors for surgical recurrence.

**Results:**

Among 3498 articles screened, 55 were included, of which 31 studies reported cumulative 5- and/or 10-year surgical recurrence rates (study inclusion period: 1932-2020; *n* = 28 354 patients). Meta-regression analysis indicated a declining trend in both 5- and 10-year surgical recurrence rates, which stabilized after 2000. In the biologics era, pooled 5- and 10-year surgical recurrence risks were 5.7% (95% CI, 3.9-8.4) and 13.0% (8.3-19.7), respectively. Smoking, patient sex, perianal disease, and behavior were not significantly prognostic for surgical recurrence after primary ICR.

**Conclusion:**

Approximately 1 in 17 patients experience surgical recurrence within 5 years after primary ICR. The risk of surgical recurrence decreased over the past decades but has stabilized in the biologics era.

**Systematic Review Registration:**

*PROSPERO registration No.* CRD42020213027.

## Introduction

Crohn disease (CD) is a chronic inflammatory disease of the gastrointestinal tract, characterized by a relapsing and remitting course, with the terminal ileum being the most commonly affected location.^1^^,^^2^

Over the past decades, the management of CD has evolved significantly, with advancements in medical therapies and monitoring strategies. The introduction of disease-modifying agents, such as immunomodulators and anti–tumor necrosis factor (TNF) therapy, has shifted treatment goals from controlling symptoms to achieving and maintaining deep mucosal remission, aiming to prevent disease progression and complications.^3^^,^^4^ Despite these advancements, a substantial proportion of CD patients still requires surgery, with ileocolic resection (ICR) being the most frequently performed procedure.^5^^,^^6^ In patients with ileal, ileocecal, or ileocolic disease, surgery is indicated in case of complicated disease (ie, strictures or fistula) and, as suggested by the results of the LIR!C trial, surgery is also a valid option for uncomplicated disease. The LIR!C trial demonstrated that laparoscopic ICR is a cost-effective alternative to infliximab treatment in patients with nonstricturing ileocecal CD, with comparable quality of life outcomes.^7^^,^^8^ Long-term follow-up has further confirmed the durability of this approach, as patients in the ICR group did not require additional surgery after a median follow-up of 5 years.^9^^,^^10^ However, surgery is not always curative in CD and postoperative disease recurrence (POR) is common.^11^ Therefore, close monitoring of inflammation through biomarkers and endoscopy has been integrated into treatment strategies to optimize outcomes.^12^ Current guidelines recommend standardized endoscopic monitoring and prophylactic medical therapy for high-risk patients to reduce POR.^13^

Previous studies demonstrated a decline in the risk of second surgery after 1980.^14^ A more recent systematic review estimated that in the 21st century, up to one-fourth of patients will require a second surgery within 10 years.^15^ However, these reviews examined all major intestinal surgeries, and contemporary estimates on the risk of surgical recurrence following ICR remain limited. We hypothesized that medical advancements, particularly in the biologics era, could lead to a decrease in surgical recurrence rates. To investigate this theory, we conducted a systematic review and meta-analysis focused exclusively on patients with Crohn’s terminal ileitis who underwent ICR. The objective of this study was to assess trends in surgical recurrence rates following primary ICR in patients with CD over the past several decades, as well as to evaluate contemporary estimates of recurrence risk and identify associated risk factors.

## Methods

A systematic review was performed to evaluate trends in surgical recurrence rates over the years following primary ICR in patients with CD by 3 independent researchers (A.E.G.H., E.D.d.W., and E.J.d.G.). The study protocol was prospectively registered at PROSPERO (registration number CRD42020213027). The present review was reported in accordance with the Preferred Reporting Items for Systematic reviews and Meta-Analyses (PRISMA) checklist (online Appendix S1**)**. The final search was performed on November 15, 2023.

### Databases and search strategy

MEDLINE (PubMed), Embase (Ovid), and the Cochrane library were searched without restrictions on publication type or publication date.

A systematic search was conducted with the assistance of an experienced clinical librarian. The following medical subject headings (MeSH) and free‐text terms were used in multiple forms and combinations: “Crohn Disease” or “Crohn” and “Digestive System Surgical Procedures” or “reoperation” or “resection” or “surgical” and “recurrence” or “surgical recurrence”. Details of the full search in all 3 databases are provided in online Appendix S2. Additional articles were identified by hand-searching the reference list of previously published systematic reviews and the included studies. No methodological filters were applied, and there were no restrictions regarding the publication dates of the included studies.

### Study selection

Studies reporting on surgical recurrence in adult CD patients following primary ileocecal or ileocolic resection were included. Included studies had to report the cumulative 5- and/or 10-year risk of surgical recurrence for inclusion in the meta-regression analysis. Studies focusing on pediatric patients or individual case reports (<30 patients undergoing primary ICR), studies written in a language other than English, and animal studies were excluded. If multiple studies reported on the same data source, the study with the most comprehensive information was selected.

All retrieved articles were separately screened on title and abstract; the full text of all potentially eligible studies was subsequently screened by 3 independent reviewers (E.J.d.G. and E.D.d.W. for the search performed in October 2019, and A.E.G.H. and E.D.d.W. for the updated search performed in 2023). Any discrepancies were re-evaluated and resolved in joint discussion. In the event of a disagreement, the opinion of a third researcher (W.A.B. or E.J.d.G.) was obtained.

### Primary and secondary outcomes

The primary outcome was the cumulative 5- and 10-year risk of surgical recurrence over the last decades. Surgical recurrence was defined as repeat resection of the neoterminal ileum or ileocolic anastomosis due to new CD-related complications. Cases requiring repeat resection within 90 days of the initial surgery, attributed to postoperative complications, were excluded.

Additionally, we aimed to estimate the contemporary risk of surgical recurrence in the biologics era. For this subanalysis, only studies including patients from the year 2000 onward were analyzed, as infliximab was first introduced in 1999.

Secondary outcomes included the effect of postoperative medical therapy following primary ICR on surgical recurrence rates, as reported in studies involving patients treated from the year 2000 onward. In addition, patient-related risk factors for surgical recurrence in the biologics era were examined.

### Data extraction and quality assessment

The 2 reviewers (A.E.G.H. and E.D.d.W.) independently extracted data from the studies. Data extraction included general study information, characteristics of included patients, and surgical recurrence rates as well as clinical and endoscopic recurrence rates and length of follow-up. For studies that did not provide numerical data but presented Kaplan–Meier curves, we extracted the required estimates and CIs.

Any disagreement was solved by consensus discussion, if necessary, with a third reviewer (E.J.d.G.). Outcome measures were quantitatively summarized and trends over time were analyzed. A separate analysis was performed for studies reporting cumulative 5-year and 10-year surgical recurrence rates to assess medium- and long-term recurrence trends.

To analyze the risk of surgical recurrence in the era of biologic therapy, studies including patients who underwent surgery from the year 2000 onward were selected. When necessary, authors of studies reporting cumulative recurrence rates were contacted up to a maximum of 3 times to provide raw data for the analysis. If no response was received within 4 weeks after the third reminder, the study was excluded from the meta-analysis.

#### Methodological assessment

In each study, the risk of bias was evaluated using the Cochrane criteria for randomized clinical trials and the Newcastle–-Ottawa Scale for cohort studies and case series.^16^^,^^17^ The Newcastle–Ottawa Scale was modified by (1) removing a redundant signaling question regarding comparability and (2) adding a signaling question regarding the median follow-up duration (1 star in case of a median follow-up >5 years).

### Statistical analysis

The pooled 5-year and 10-year risk of surgical recurrence following primary ICR was estimated using a random-effects meta-analysis model. If studies did not report sufficient information to be included in the meta-analysis, we contacted authors to retrieve these data or we calculated CIs ourselves using the Greenwood method, based on other information reported in the study. The meta-analysis model consisted of a restricted maximum likelihood estimator and Jackson's modification of the Hartung–Knapp/Sidik–Jonkman variance correction, which is superior compared to conventional models in controlling the type I error rate at 5%. A random-effects model was prespecified given the expected between-study variation in the clinical characteristics of patients. Estimates and CIs were logit-transformed prior to the meta-analysis to approximate a normal distribution, and pooled estimates were subsequently back-transformed to the original scale. Heterogeneity was assessed using the *I*^2^ and *τ*^2^ and their 95% CIs.

Additionally, meta-regression analyses were performed to assess trends in surgical recurrence over time, while accounting for the sample size of included studies. Meta-(regression-)analyses were performed in R, version 4.4.2 (R Foundation for Statistical Computing) using the metafor package.

Two sensitivity analyses were performed, excluding (1) the study by King et al.,^62^ which included 12 000 patients, and (2) studies with a fair or poor score based on the modified Newcastle–Ottawa Scale. The latter sensitivity analysis was only performed for the meta-regression analyses, as none of the studies included in the overall meta-analysis had risk of bias concerns.

For the secondary analysis assessing risk factors for surgical recurrence, studies reporting univariable (“unadjusted”) hazard ratios (HRs) were included for a pooled analysis, in which HRs were pooled after log-transformation using a random-effects model with a restricted maximum likelihood estimator and Jackson’s modification of the Hartung–Knapp/Sidik–Jonkman variance correction. Pooled log-transformed HRs were subsequently exponentiated to derive the pooled HR. Studies reporting adjusted HRs were included in the forest plot for reference but were not incorporated into the pooled analysis, given the expected variation between studies in terms of the variables that were adjusted for in the multivariable models. Studies reporting odds ratios (ORs) were not included in the pooled analysis, as ORs are inappropriate for time-to-event outcomes such as surgical recurrence.

## Results

### Literature search and study selection

**Figure 1:**
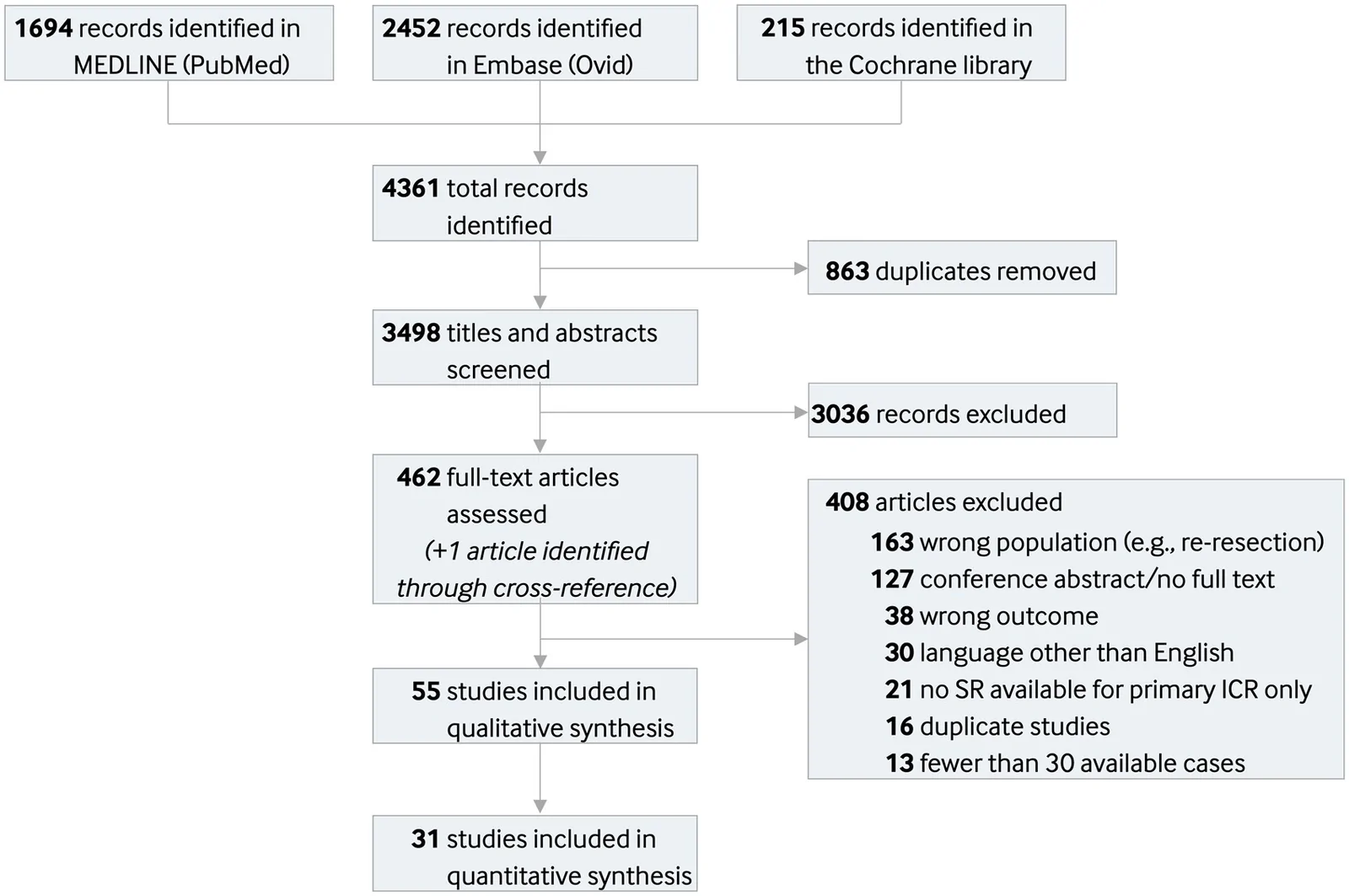
Preferred Reporting Items for Systematic reviews and Meta-Analyses (PRISMA) flowchart.

The literature search identified a total of 4361 studies; after removal of duplicates, the titles and abstracts of 3498 unique studies were screened (Figure 1). Following full-text screening of 462 potentially eligible articles, 55 studies met the inclusion criteria and reported surgical recurrence rates.^5^^,^^9^^,^^18–70^ Of these, 7 studies reported only cumulative recurrence rates,^5^^,^^52^^,^^53^^,^^59^^,^^64^^,^^67^^,^^70^ 24 studies reported only overall surgical recurrence rates,^18^^,^^21^^,^^24^^,^^28–30^^,^^32^^,^^35^^,^^36^^,^^38^^,^^39^^,^^43–50^^,^^56^^,^^60^^,^^61^^,^^63^^,^^65^ and 24 studies reported both cumulative and overall recurrence rates.^9^^,^^19^^,^^20^^,^^22^^,^^23^^,^^25–27^^,^^31^^,^^33^^,^^34^^,^^37^^,^^40–42^^,^^51^^,^^54^^,^^55^^,^^57^^,^^58^^,^^62^^,^^66^^,^^68^^,^^69^ Consequently, a total of 31 studies provided cumulative recurrence rates and were included in the meta-regression analysis to assess surgical recurrence rates over time.^5^^,^^9^^,^^19^^,^^20^^,^^22^^,^^23^^,^^25–27^^,^^31^^,^^33^^,^^34^^,^^37^^,^^40–42^^,^^51–55^^,^^57–59^^,^^62^^,^^64^^,^^66–70^

Nine out of 13 studies conducted in the biologics era (after the year 2000) were eligible for inclusion in the meta-analysis to estimate the pooled risk of surgical recurrence in this era. All studies included in the analysis were cohort studies, of which 7 were conducted prospectively and the rest retrospectively.

### Study characteristics

Details of the 31 included studies are presented in Table 1 for those reporting cumulative recurrence rates, and in Table S1 (online Appendix S2) for the 48 studies reporting overall surgical recurrence rates. The included studies span an inclusion period from 1932 to 2019.^5^^,^^9^^,^^18–70^ The study sample sizes varied from 41 to 12 230 patients,^27^^,^^62^ with a combined total of 28 354 patients across all studies. Most studies were conducted in Europe or North America.

**Table 1:** Characteristics of included studies reporting cumulative surgical recurrence rates.

Study first author, publication year	Country, study design	Study span, y	Sample size (primary ICR)	Follow-up duration after ICR, y	Age at surgery, y^a^	Sex, male(%)	Smoker, yes (%)	Disease behavior	Disease location	Medication preoperative (primary ICR), *n* (%)	Medication postoperative following primary ICR, *n* (%)	Cumulative surgical recurrence
**Lennard-Jones^19^, 1967**	UK, retro MC cohort	1947-1965	71	NR	Mean 36 (range 16-23)	42 (59)	NR	NR	NR	NR	NR	5-y: 16% 10-y: 26%
**Alexander-Williams^20^, 1972**	UK, retro SC cohort	1950-unkown	89	Mean 14 (range 4-38)	NR	NR	NR	NR	NR	NR	NR	5-y: 26.5% 10-y: 50%
**Homan^22^, 1978**	US, retro SC cohort	1932-1975	102	NR	Mean 33 (SD NR)	NR	NR	NR	NR	CS: 26 (26)	NR	5-y: 17% 10-y: 42% 15-y: 65%
**Higgens ^23^, 1980**	UK, retro SC cohort	1944-1979	180	NR	NR	NR	NR	NR	L1: 180 (100)	NR	NR	5-y: 17.6% 10-y: 36% (95%CI 24-48) 15-y: 51% (95% CI 37-65)
**Whelan^25^, 1985**	US, retro SC cohort	1966-1969	225	Mean 13^a^ (SD NR)	NR	224^b^ (52)	NR	NR	NR	NR	NR	5-y: 40% 10-y: 60%
**Adlof^26^, 1987**	France, retro SC cohort	1972-1983	46	Range 3-15 Mean or median NR	Mean 32 (range 13-61)	26 (45)	NR	NR	L1: 15 (33) L3: 31 (67)	NR	NR	5-y: 22% 10-y: 58%
**Kangas^27^, 1987**	Finland, retro MC cohort	1962-1985	41	Median 8 (range <1-23)	Median 35^b^ (range 15-70)	35^b^ (48)	NR	NR	NR	NR	NR	5-y: 21%
**Nordgren^31^, 1994**	Sweden, pros SC cohort	1968-1977	68	Median 17^a^ (IQR 15-18)	Median 27^b^ (IQR 23-34)	NR	NR	NR	L1: 68 (100)	NR	NR	5-y: 23% 10-y: 35% 15-y: 45%
**Sachar^34^, 1996**	US, SC	1961-1984	Fist: 35	Median 9 (IQR NR)	Median 22 (IQR NR)	21 (60)						5-y: 12% 10-y: 30%
			Stenotic: 36		Median23 (IQR NR)	14 (38)						5-y: 9% 10-y: 18%
**Poggioli^33^, 1996**	Italy, pros SC cohort	1978-1993	157	Range 2-16 yr Mean or median NR	Mean^b^ 36 ± 1	120^b^ (44)	NR	NR	NR	NR	NR	5-y: 10% 10-y: 39% 20-y: 61%
**Yamamoto^37^, 1999**	UK, MC	1975-1995	Perf: 72	Median 7 (range < 1-23)	Median 29 (range 13-71)	26 (36)	33 (46)	B3: 72 (100)			CS: 28 (39) 5-ASA: 10 (14)	5-y: 22% 10-y: 39%
			Non-perf: 93		Median 29 (range 11-69)	31 (33)	44 (48)	B1/B2: 93 (100)			Steroids: 38 (41) 5-ASA: 20 (22)	5-y : 27% 10-y: 45%
**Lowney^40^, 2006**	US, retro SC cohort	1987-2003	LICR: 63	Mean 5 (SD NR)	Median 35 (range 18-76)	26 (41)	NR	NR	NR	CS: 29 (46) Imm: 15 (24)	Chemoproph: 25 (39)	5-y: 5%
			OICR: 50	Mean 7 (SD NR)	Median 37 (range 17-78)	17 (34)	NR	NR	NR	CS: 25 (50) Imm: 13 (26)	Chemoproph: 27 (54)	5-y: 17.5%
**Cullen, ^41^** **2007**	Ireland, retro SC cohort	1980-2000	139	Median 7^a^ (IQR 5-11)	Median 29 (IQR 24-35)	55 (40)	68 (49)	B1: 88 (63) B2: 18 (13) B3: 33 (24)	NR	NR	NR	5-y: 17% 10-y: 35% 15-y: 50%
**Eshuis^42^, 2008**	NL, retro MC cohort	1995-1998	OICR: 44	Median 9 (range 4-10)	NR	10 (23)	21 (48)	NR	NR	NR	Proph: TP: 8 (18) MTX: 1 (2) 5-ASA: 13 (30)	5-y: 15
			LICR: 27	Median 9 (range 6-10)	NR	7 (26)	14 (52)	NR	NR	NR	Proph: 5-ASA: 12 (44)	5-y: 16%
Riss^51^, 2014	Austria, retro SC cohort	1997-2006	116	Mean 8 (±3)	Mean 33 (±12)	66 (57)	38 (33)	NR	NR	NR	Bio (anti-TNF): 13 (11) AZA/MTX: 56 (48)	5-y: 3.6% 10-y: 12%
**De Buck van Overstraeten ^53^, 2017**	Belgium, retro MC cohort	1998-2013	538	Median 6 (IQR 2-9)	Median 31 (IQR 24-42)	215 (40)		B1: 37 (7) B2: 270 (50) B3: 231 (43)	L1: 366 (68) L3: 172 (32)	CS: 278 (52) Bio (anti-TNF): 111 (21) AZA: 218 (41) MTX: 25 (5)	NR	5-y: 6.5% 10-y: 19.1%
**Li^52^, 2015**	US, pros SC cohort	2000-2012	Non-transfused: 266	Mean 6 (±4)	Mean 36 (±14)	111 (42)	68 (26)	B1: 30 (11) B2: 109 (41) B3: 127 (48)	NR	CS: 173 (65) Imm: 63 (24) Bio (anti-TNF): 58 (22)	5-ASA: 69 (26) Imm: 79 (30) Bio (anti-TNF): 28 (11)	5-y: 12% 10-y: 25%
			Transfused: 52	Mean 5 (±1)	Mean 37 (±17)	26 (50)	14 (27)	B1: 6 (12) B2: 15 (29) B3: 31 (60)		CS: 28 (54) Imm: 12 (23) Bio (anti-TNF): 21 (40)	5-ASA: 18 (35) Imm: 14 (27) Bio (anti-TNF): 5 (10)	5-y: 35% 10-y: 42%
**Guo^55^, 2018**	China, retro SC cohort	2006-2015	166	Mean 4 (±2)	Mean 37 (±11)	112 (67)	22 (13)	B1: 8 (5) B2: 64 (39) B3: 94 (57)	L1: 46 (28) L3: 105 (63)	CS: 6 (4) Bio (anti-TNF): 13 (8) AZA: 68 (41)	Proph: AZA: 91 (54.8)	5-y: 13.6%
**Bolckmans^58^, 2019**	UK, retro MC cohort	2000-2012	C1: 305 (one-stage)	Median 9 (IQR 6-12)	Median 35 (IQR 26-46)	141 (46)	60 (20)	B3: 85 (28)	L1: 164 (54) L3: 133 (44)	NR	Proph: 125/242 (54) Bio (anti-TNF/VDZ): 41 (17) TP: 60 (25) MTX: 2 (<1)	5-y: 5.4% (95% CI: 3.1-7.7) 10-y: 19.8% (95% CI: 14.4-24.8)
			C2: 77 (2-stage)	Median 8 (IQR 7-10)	Median 33 (IQR 24-45)	45 (58)	25 (32)	B3: 40 (52)	L1: 45 (58) L3: 32 (42)	NR	Proph: 42/72 (58) Bio (anti-TNF/VDZ): 7 (11) TP: 20 (25)	
**Coffey^54^, 2019**	Ireland, retro SC cohort	2004-2010	MS: 30	Mean 6 (±4)	38 (±14)	14 (47)	20 (67)	B1: 16 (53) B2: 6 (20) B3: 8 (27)	L1: 23 (77) L3: 5 (17)	CS: 13 (43) Imm: 11 (37) Bio (NS): 5 (17)	Proph: TP: 4 (13) Bio (anti-TNF): 2 (7)	5-y: 40%
		2010-NR	EM: 34	Mean 4 (±2)	36 (±12)	14 (41)	20 (59)	B1: 8 (24) B2: 14 (41) B3: 12 (35)	L1: 26 (76) L3: 6 (18)	CS: 12 (35) Imm: 10 (29) Bio (NS): 15 (44)	Proph: TP: 4 (12) Bio (anti-TNF): 4 (12)	5-y: 2.9%
Coletta^57^, 2019	Italy, retro SC cohort	2004-2016	118	Median 5 (range < 1-13)	NR	64 (54)	47 (40)	B1: 12 (10) B2: 63 (53) B3: 43 (36)	L1: 105 (89) L3: 13 (11)	CS: 4 (3) TP + Bio (anti-TNF):71 (60)	5-ASA: 40 (34) CS: 37 (31) TP + Bio (anti-TNF): 38 (32)	5-y: 8% 10-y: 14%
**Stevens^9^, 2020**	NL, retro MC cohort	2008-2015	69	Median 5 (IQR 3-8)	Mean 33 (±14)	24 (35)	21 (30)	B1: 96 (100)	L1: 69 (100)	NR	Imm: 26 (38)	5-y: 0% (95%CI: 0.0-9.6)
**Kalman^59^, 2020**	Sweden, National registry	1990-1995	1315	NR	NR	NR	NR	NR	NR	NR	NR	5-y: 5.5% (95% CI: 4.3-6.8) 10-y: 13.0% (95% CI: 11.1-14.9)
		1996-2000	1087	NR	NR	NR	NR	NR	NR	NR	NR	5-y: 4.4% (95% CI: 3.1-5.6) 10-y: 8.8% (95% CI: 7.0-10.6)
		2001	282	NR	NR	NR	NR	NR	NR	NR	NR	5-y: 3.5% (95% CI: 1.2-5.6) 10-y: 7.3% (95% CI: 3.8-10.6)
		2002-2008	1163	NR	NR	NR	NR	NR	NR	NR	NR	5-y: 3.2% (95% CI: 2.1-4.3) 10-y: 5.3% (95% CI: 3.7-6.8)
		2009-2014	613	NR	NR	NR	NR	NR	NR	NR	NR	5-y: 2.2% (95% CI: 0.5-3.8)
**Beelen^5^, 2021**	NL, PALGA-database	1991-2015	3186	Median 9^a^ (IQR 4-15)	NR	NR	NR	NR	NR	NR	NR	10-y: 6.7%
**King^62^, 2021**	UK, NHS Hospital statistics database	2007-2016	12.230	Median 6 (IQR 4-9)	Median 36 (IQR 26-50)	5475 (44.8)	NR	NR	NR	NR	NR	5-y: 9% (95% CI: 8.4-9.6) 10-y: 16.9% (95% CI: 15.3-18.5)
**Mineccia^64^, 2022**	Italy, MC	2009-2019	326 EM: 204 MS: 122	Mean 5 (±3)	EM: Mean 41 (±15)	107 (33)	110 (34)	B2: 107 (33) B3: 219 (67)	NR	5-ASA: 160 (49) CS: 62 (19) Imm: 34 (10) Bio (NS): 48 (16)	Proph: Imm and/or Bio (IFX/adA/VDZ): 180 (55)	5-y: 4% (95% CI: 2.7-5.3)
					MS: Mean 4 (±16)							
**Carvello^68^, 2023**	Italy, retro MC cohort	2008-2018	262 Non-R: 117	Median 5 (IQR 2-7)	Mean 34 (±13)	137 (52)	80 (31)	B1: 70 (27) B2: 94 (36) B3: 98 (37)	L1: 144 (55) L3: 105 (40)	CS: 177 (68) Bio (anti-TNF): 109 (42) Monoclonal antibodies: 25 (10)	Proph: 43 (16), not further specified.	5-y: 6% (95% CI: 2-11) 10-y: 35% (95% CI: 17-63)
			R: 145	Median 5 (IQR 3-7)	Mean 36 (±14)							
**Bislenghi^67^, 2023**	Belgium, SC	2005-2013	C1. Handsewn E-E: 51	Median 14 (IQR 10-16)	Median 33 (IQR 24-46)	16 (31)	8 (16)	B1: 1 (2.0) B2: 21 (41) B3: 29 (57)	NR	CS: 16 (31) Bio (NS): 11 (22)	Proph: CS: 1 (2) Imm: 9 (18) Bio (NS): 1 (2)	5-y: 10.6% (95% CI: 4.5-23.6) 10-y: 17.7% (95% CI: 9.2-32.4)
			C2. Stapled S-S: 76	Median 9 (IQR 8-10)	Median 31 (IQR 24-47)	36 (47)	17 (22)	B1: 0 (0) B2: 42 (55) B3: 34 (45)	NR	CS: 14 (18) Bio (NS): 20 (26)	Proph: CS: 0 (0) Imm: 5 (7) Bio (NS): 2 (3)	5-y: 0% (ND; ND) 10-y: 6.5% (1.9-19.9)
**Assaf^66^, 2023**	Israel, retro SC cohort	2010-2021	189	Median 6 (range 1-1.5[sic]	Mean 34 (range 15-78)	104 (55)	40 (21)	B2: 71 (38) B3: 76 (40) B2 + B3: 42 (22)	L1: 126 (67) L3: 61 (32)	CS: 26 (14) Bio (NS): 36 (19)	CS: 9 (5) TP: 44 (23) Bio (NS): 88 (47)	5-y: 2.5%
**M. Bak^70^, 2023**	NL, MC	2000-2019	652	Mean 6 (±5)	Mean 36 (±14)	242 (37)	231 (35)	B1: 149 (23) B2: 323 (50) B3: 180 (28)	L1: 416 (64) L3: 236 (36)	CS: 536 (82) Imm: 433 (66) Bio: 315 (48) - anti-TNF: 294 (45) - UST: 7 (1) - VDZ: 14 (2)	Proph; Imm: 146 (22) Bio: 58 (9) - anti-TNF: 52 (22) - UST: 5 (2) - VDZ: 1 (0) Imm + Bio (anti-TNF): 35 (5)	5-y: 15% (95% CI: 11-19) 10-y: 22% (95% CI: 18-26)
**Aratari^69^, 2024**	Italy, MC	C1: 1980-1998	198	Mean 9 (±3)	Median 30 (IQR 24-39)	117 (59)	77 (39)	B1/B2: 131 (66) B3: 67 (34)	NR	5-ASA/AB: 125 (77) CS: 46 (23) Imm: 2 (1) Bio: -	5-ASA/AB: 135 (68) CS: 40 (20) Imm: 11 (6) Bio (anti-TNF):1 (<1)	5-y: 7.1% (95% CI: 3.9-11.6) 10-y: 19.7% (95% CI: 14.4-25.9)
		C2: 1999-2010	218	Mean 8 (±3)	Median 37 (IQR 28-48)	108 (50)	87 (40)	B1/B2: 144 (66) B3: 74 (34)	NR	5-ASA/AB: 119 (56) CS: 73 (34) Imm: 28 (13) Bio (anti-TNF): 13 (6)	5-ASA/AB: 146 (67) CS: 57 (26) Imm: 92 (42) Bio (anti-TNF): 60 (28)	5-y: 6.4% (95% CI: 3.6-10.5) 10-y: 11.9% (95% CI: 7.9-17.0)
		C3: 2011-2020	241	Mean 5 (±3)	Median 45 (IQR 33-57)	123 (51)	120 (50)	B1/B2: 180 (75) B3: 61 (25)	NR	5-ASA/AB: 87 (36) CS: 102 (42) Imm: 66 (27) Bio (anti-TNF): 75 (31)	5-ASA/AB: 139 (58) CS: 31 (13) Imm: 60 (25) Bio (anti-TNF): 124 (52)	5-y: 4.6% (95% CI: 2.3-8.0) 10-y: 7.9% (95% CI: 4.8-12.0)

Abbreviations: 5-ASA, mesalazine; AB, antibiotic; ADA, adalimumab; anti-TNF, anti-tumour necrosis factor; B1, inflammatory; B2, fibrostenotic; B3, penetrating; Bio, biologicals; CS, corticosteroids; EM, extended mesenteric excision; fist, fistulizing disease; IFX, infliximab; Imm, immunomodulator; L1, ileal; L2, colonic; L3, ileocolonic; LICR, laparoscopic ileocolic resection; MC, multicenter; MS, mesenteric-sparing excision; Non-R, nonrecurrent; non-perf, nonperforated disease; NR, not reported; NS, not specified; OICR, open ileocolic resection; perf, perforated disease; proph, prophylactic; pros, prospective; R, recurrent; retro, retrospective; SC, single center; TP, thiopurines; UK, United Kingdom; US, United States; UST, ustekinumab; VDZ, vedolizumab.

a Follow-up for the entire cohort and not specifically for those patients who underwent a primary ileocecal resection.

b Variable for the entire cohort and not specifically for those patients who underwent a primary ileocecal resection.

Clinical features of CD are summarized in Table 1 for studies reporting cumulative recurrence rates.^5^^,^^9^^,^^19^^,^^20^^,^^22^^,^^23^^,^^25–27^^,^^31^^,^^33^^,^^34^^,^^37^^,^^39–41^^,^^51–55^^,^^57–59^^,^^62^^,^^64^^,^^66–70^ Disease behavior was reported in 16 studies,^9^^,^^22^^,^^37^^,^^41^^,^^52–55^^,^^57^^,^^58^^,^^64^^,^^66–70^ and information on disease location was documented in 13 studies.^9^^,^^23^^,^^26^^,^^31^^,^^34^^,^^53–55^^,^^57^^,^^58^^,^^66^^,^^68^^,^^70^ Seventeen studies provided information on pre- and postoperative medication use.^9^^,^^22^^,^^37^^,^^40^^,^^42^^,^^51^^,^^52^^,^^54^^,^^55^^,^^57^^,^^58^^,^^64^^,^^66–70^

Cumulative surgical recurrence rates were reported as follows: 30 studies assessed 5-year risk of surgical recurrence, 24 studies reported 10-year risk, 4 studies also reported 15-year risk, and 1 study reported the 20-year risk of surgical recurrence.

Thirteen studies reported data exclusively from patients who underwent primary ICR in the pre-biologics era, ie, prior to the year 2000.^18^^,^^20^^,^^22^^,^^23^^,^^25–27^^,^^31^^,^^33^^,^^34^^,^^37^^,^^41^^,^^42^

#### Risk of bias

A summary of the risk of bias assessment is provided in Table S2 (online Appendix S2). Since only cohort studies met the inclusion criteria, the risk of bias was evaluated with the Newcastle–Ottawa Scale. Among the 55 cohort studies, 91% (*n* = 50) were rated as having overall good quality and one study (2%) rated as having fair quality. In contrast, 4 studies were considered to have overall poor quality.

### Risk for surgical recurrence

#### Surgical recurrence over time

**Figure 2:**
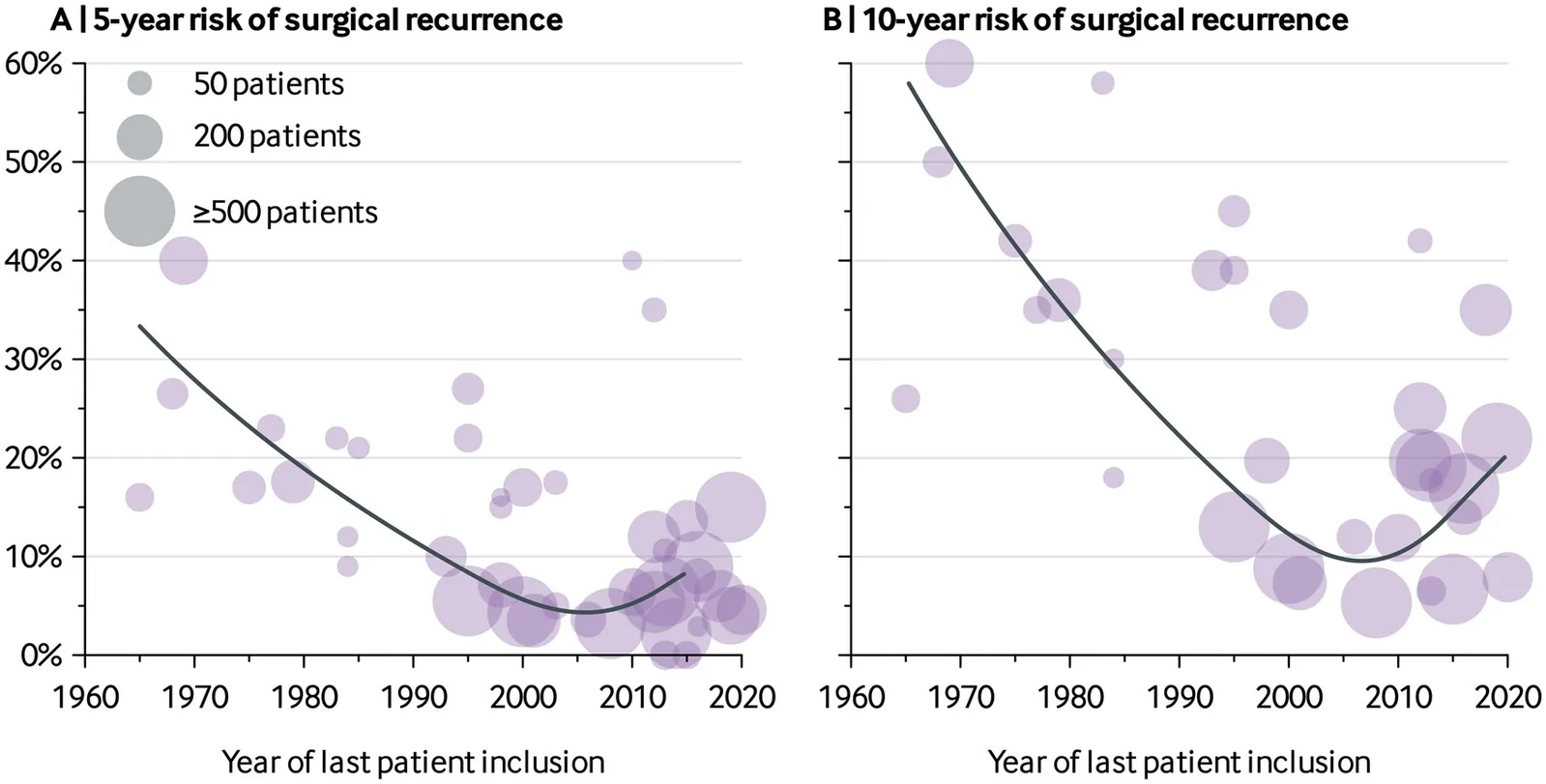
Cumulative 5- and 10-year risk of surgical recurrence over time. Each point represents a study, with the year of last patient inclusion used as the reference point in the graph. The legend (gray circles) serves as a reference for study sample sizes for both figures, with example markers for 50, 200, and ≥500 patients.

Cumulative surgical recurrence rates were reported in 31 studies, comprising 25 209 patients. Cumulative surgical recurrence rates ranged from 0% to 40% at 5 years and from 5% to 60% at 10 years postoperatively. The 5- and 10-year risk of surgical recurrence in CD following ICR over the past decades is illustrated in Figure 2. Meta-regression analyses indicate a declining trend in both 5- and 10-year surgical recurrence rates over time. Around the year 2000, when biologicals were introduced, the decline stabilizes followed by a slight increase in the 10-year surgical recurrence rate. The same trend was also seen in a sensitivity analysis excluding the study by King et al.^62^ (2021), which accounted for >50% of all included patients in the meta-regression analysis (Figure S1, online Appendix S2), and a sensitivity analysis excluding all studies which were scored as “fair” or “poor” based on the Newcastle-Ottawa scale (Figure S2, online Appendix S2).

#### Risk of surgical recurrence in the biologics era

**Figure 3:**
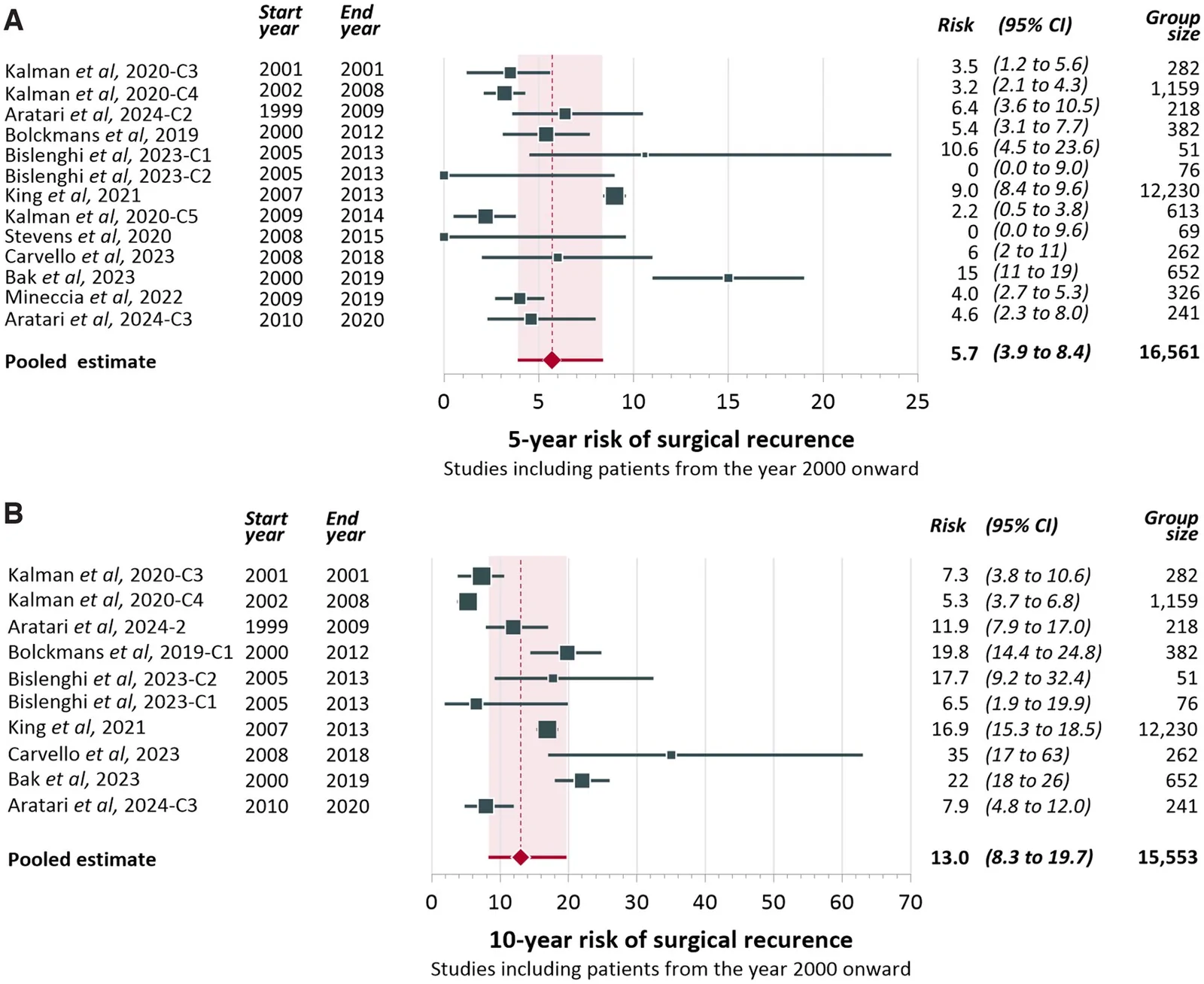
(A) Cumulative risk of surgical recurrence by 5 years after primary ICR. Studies providing 95% CIs are included. Studies are arranged in order of last inclusion year. (B) Cumulative risk of surgical recurrence by 10 years after primary ICR. Studies providing 95% CIs are included. Studies are arranged in order of last inclusion year.

Thirteen studies reported cumulative 5-year and/or 10-year surgical recurrence rates of patients operated on during the biologics era.^9^^,^^52^^,^^54^^,^^55^^,^^57–59^^,^^62^^,^^64^^,^^66–70^ Of these, 9 studies were eligible for meta-analysis, comprising 96% of the potentially eligible 20.101 patients; 2 studies provided sufficient data directly, and additional information was obtained for 7 studies through author correspondence.^9^^,^^58^^,^^59^^,^^62^^,^^64^^,^^67–70^ All 13 studies included in the meta-analysis were assessed and found to have overall good methodological quality. For patients who underwent ICR from 2000 onward, the pooled risk of surgical recurrence is presented in Figure 3. The pooled 5-year cumulative risk of surgical recurrence, based on nine studies including 16 561 patients, was 5.7% (95% CI, 3.9 to 8.4%) with considerable between-study heterogeneity (*I*^2^, 89% [73% to 97%], *P* < .0001).^9^^,^^58^^,^^59^^,^^62^^,^^64^^,^^66–69^ The pooled 10-year cumulative risk of surgical recurrence (*n* = 7 studies, 15 553 patients) was 13.0% (95% CI, 8.3%-19.7%) with high between-study heterogeneity (*I*^2^, 93% [83%-98%], *P* < .0001).^58^^,^^59^^,^^62^^,^^67–70^ There were 2 studies, including the retrospective long-term follow-up of the randomized LIR!C trial, that reported a 0% 5-year surgical recurrence rate.^9^^,^^67^

#### Sensitivity analysis

Given that the study by King et al.^62^ accounted for up to 74% and 78% of patients included in the meta-analysis of 5- and 10-year surgical recurrence rates, respectively, a leave-one-out sensitivity analysis was performed to evaluate the robustness of the results. However, results were not materially affected by excluding the study of King et al. with pooled 5-year and 10-year surgical recurrence rates of, respectively, 5.4% (95% CI, 3.5%-8.2%) and 12.5% (7.5%-20.1%).

### Perioperative treatment regimen

Five studies conducted primarily in the biologics era investigated the effect of various postoperative treatment strategies on surgical recurrence in CD.^51^^,^^53^^,^^55^^,^^64^^,^^69^ A formal meta-analysis to estimate pooled HRs was not deemed feasible due to substantial heterogeneity in treatment regimens and timing of postoperative initiation. A multicenter study found that immediate postoperative therapy (including steroids, anti-TNF agents, azathioprine [AZA], aminosalicylates, and methotrexate) did not significantly reduce the risk of surgical recurrence compared with delayed or no treatment (HR, 0.82 [95% CI, 0.46-1.45]).^53^ In contrast, Guo et al. reported that both immediate and endoscopy-driven treatments with AZA were associated with a lower surgical recurrence risk compared with symptom-driven treatment (HR, 0.45 [0.22-0.95] and HR, 0.22 [0.13-0.83], respectively).^55^ Consistent with these findings, another study showed a protective effect of postoperative AZA (AZA/6-MP) use for more than 3 months in reducing surgical recurrence risk.^51^ In addition, Mineccia et al. also found that postoperative adjuvant treatment for at least 6 months with immunosuppressive or biologic agents (infliximab, adalimumab, or vedolizumab) could reduce surgical recurrence (adjusted HR, 0.5 [0.48-0.55]).^64^ Aratari et al. found that early postoperative use (within 1 year after surgery) of immunomodulators (IMMs) and/or anti-TNF agents did not significantly reduce surgical recurrence compared to therapy initiated later in the postoperative disease course.^69^ Similarly, in this study no difference was observed in prophylactic (immediately post-surgery) and reactive (after endoscopy within 1 year) strategies (HR, 1.07 [0.39-2.95]).

### Risk factors for surgical recurrence

Among the 55 included studies, 28 involved patient cohorts mainly operated on in the biologics era. Of these, 8 studies reported potential risk factors along with corresponding HRs for surgical recurrence following primary ICR and were included in this subanalysis.^51–55^^,^^62^^,^^64^^,^^69^

#### Active smoking

**Figure 4:**
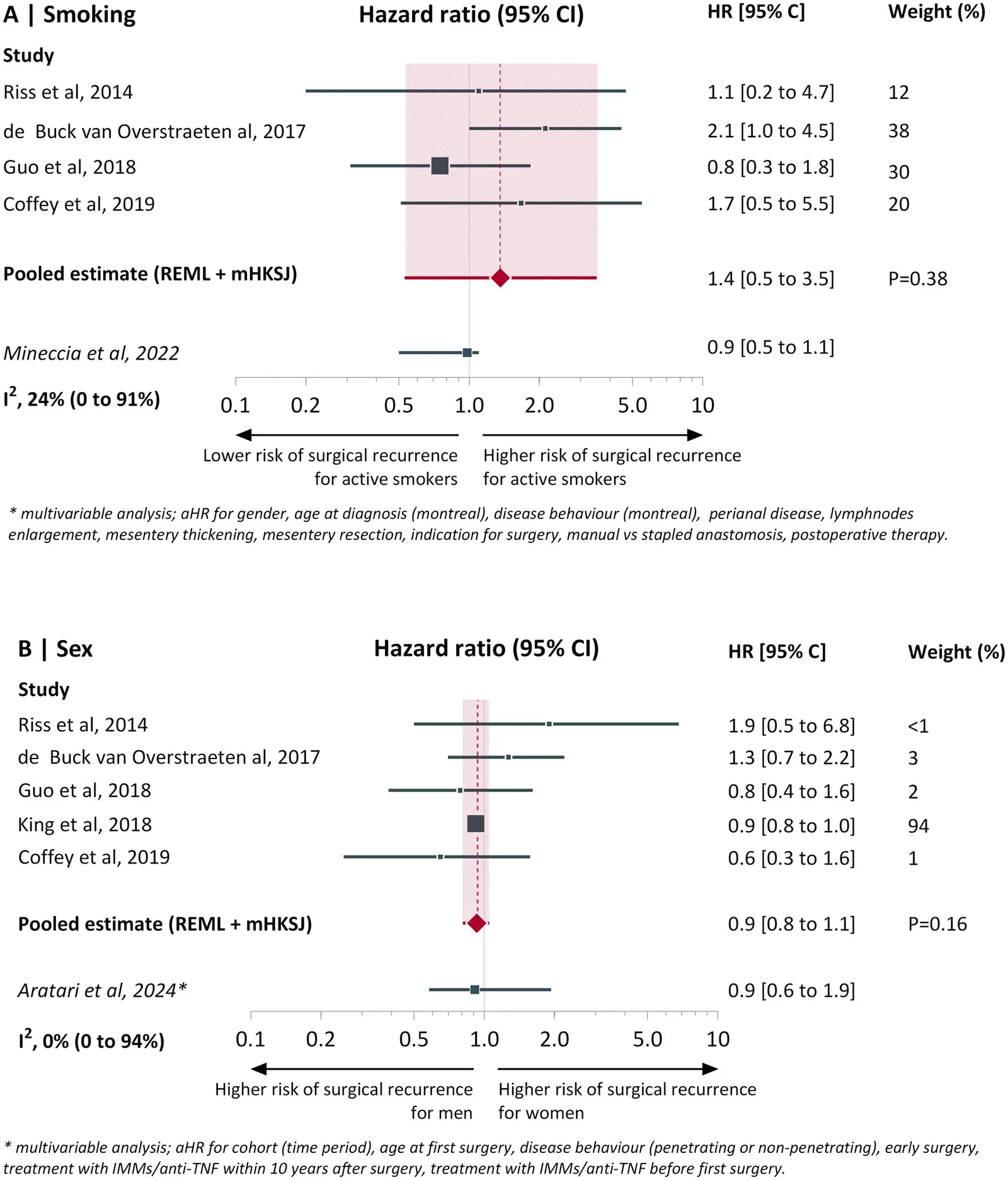
Forest plot showing the association of (A) smoking and (B) sex with surgical recurrence following primary ICR in CD.

Seven studies examined the association between active smoking at the time of surgery and surgical recurrence after primary ICR, of which 5 are shown in the forest plot.^47^^,^^49^^,^^51^^,^^53–53^^,^^64^ Active smoking was uniformly defined as smoking at the time of surgery, without specifying daily cigarette consumption. The pooled, unadjusted HR for active vs nonactive smoking was 1.36 (95% CI, 0.53-3.52; Figure 4A), with no evidence for between-study heterogeneity (*I*^2^, 24% [0%-91%]). After multivariable adjustment, Mineccia et al. reported an adjusted HR of 0.9 (0.5-1.1).

**Figure 5:**
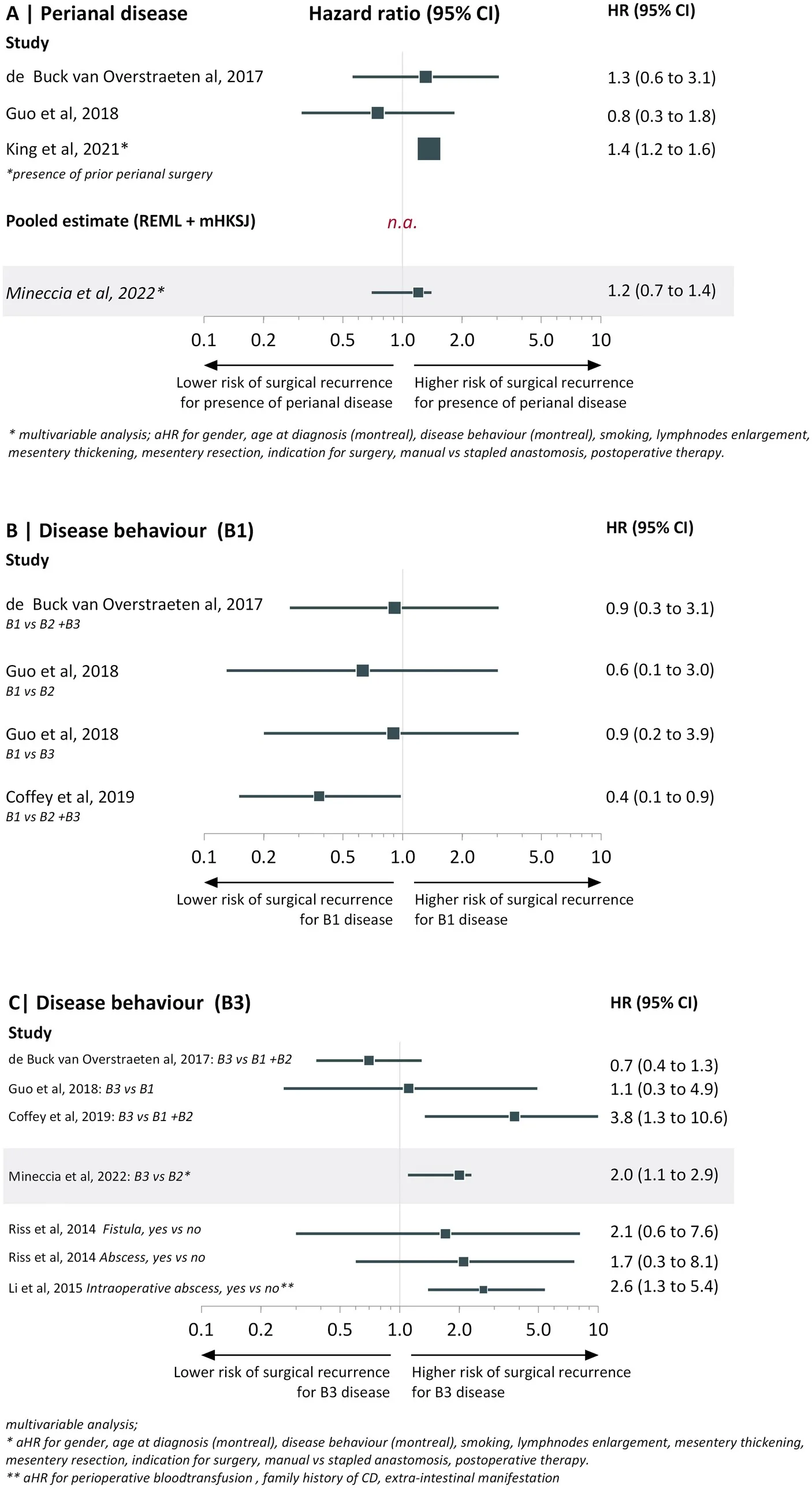
Forest plot illustrating the association between patient characteristics and surgical recurrence following primary ICR for CD): (A) perianal disease, (B) nonstricturing, nonpenetrating disease (B1), and (C) penetrating disease behavior. *Note: No meta-analysis was performed*.

#### Sex

The association of sex with surgical recurrence was evaluated in 8 studies, of which 5 studies (13 114 patients) provided enough data to perform meta-analyses.^47^^,^^49^^,^^51^^,^^53–55^^,^^62^^,^^69^ The pooled, unadjusted HR for women vs men was 0.93 (0.81 to 1.05; I^2^, 0% [0 to 94%], Figure 4B). Aratari et al. additionally investigated the adjusted difference in the risk of surgical recurrence between men and women, reporting an adjusted HR of 0.91 (0.58-1.93).^69^

#### Other risk factors

Several other risk factors and their prognostic value for surgical recurrence were described (ie, disease behavior, disease location, perianal disease, age at diagnosis, age at surgery, and disease duration at surgery). These risk factors were not assessed in a sufficient number of studies to formally perform meta-analyses and estimate pooled HRs. Similar variables with their corresponding HRs are shown in Figure 5. However, none of the risk factors were clearly associated with surgical recurrence, due to either (1) large clinical and statistical between-study heterogeneity, or (2) insufficient sample sizes with a low number of events, resulting in wide CIs and insufficient statistical power to detect potential risk factors.

##### Disease behavior, disease location, perianal disease

The association between disease behavior and surgical recurrence varied between studies.^51^^,^^53–53^^,^^64^ While 2 studies reported no significant association,^52^^,^^55^ others observed a risk of recurrence risk with penetrating (B3) disease.^49^^,^^64^ One study found a lower risk with B1 disease (HR, 0.38 [0.15-0.98]) and higher risk with B3 disease (HR, 3.78 [1·35-10·64]), though these were not significantly prognostic after multivariable adjustment.^54^ Similarly, B3 disease was associated with recurrence in multivariable analyses in another study (adjusted hazard ratio [aHR] 2.0 [1.1-2.3]).^64^ Intraoperative findings (abscesses, fistulas, stenosis) showed no consistent association with recurrence.^51^^,^^52^ Only 1 study examined disease location in relation to surgical recurrence, with no significant association.^55^ Four studies assessed perianal disease as a risk factor.^53^^,^^55^^,^^62^^,^^64^ Only 1 study identified a significant association, reporting that prior perianal surgery increased the risk of surgical recurrence.^61^

##### Age at diagnosis, age at surgery, and disease duration at surgery

Six studies examined age at diagnosis, age at surgery, and disease duration in relation to surgical recurrence.^51^^,^^53^^,^^55^^,^^62^^,^^64^^,^^69^ Guo et al. found that patients diagnosed at age ≤16 years (A1) had a higher recurrence risk than those diagnosed at 17-40 years (A2), findings significant in both univariable (HR, 3.6 [1.2-10.4]) and multivariable analysis (aHR, 3.4 [1.2-9.8]).^55^ Other studies found no association with age at diagnosis,^64^^,^^69^ age at surgery,^51^^,^^54^^,^^61^ or disease duration.^53^^,^^55^

## Discussion

This systematic review and meta-analysis demonstrates a decline in surgical recurrence rates at 5 and 10 years postoperatively in patients undergoing primary ICR for CD over the past 87 years. Contrary to our expectations, there was no clear further reduction in recurrence rates observed following the introduction of anti-TNF. In the biologics era, the contemporary 5- and 10-year risk of surgical recurrence following primary ICR are estimated at 5.7% and 13.0%, respectively. These trends were not materially impacted in a sensitivity analysis excluding the largest study and a sensitivity analysis excluding studies with a potential risk of bias.

The current 5- and 10-year recurrence rates of respectively 5.7% and 13.0% are notably lower than those reported in earlier systematic reviews.^14^^,^^15^ For example, Frolkis et al.^14^ estimated a 10-year risk of repeat surgery of 33.2% in cohorts studied after 1980, while a more recent review focusing on patients who underwent surgery in the 21^st^ century reported 5- and 10-year risks of 14.8% and 25.5%, respectively.^15^ Our review includes more contemporary studies and specifically focused on patients undergoing primary ICR, rather than broader surgical populations, which may have contributed to the lower observed surgical recurrence rates. Interestingly, 2 studies in the present review reported a 0% 5-year surgical recurrence rate,^9^^,^^67^ including the long-term follow-up study of the randomized LIR!C trial, which compared laparoscopic ICR with infliximab for terminal ileitis in CD. None of the patients in the ICR treatment arm required surgery for POR within 5 years. Variability in surgical practices, such as the higher rate of surgery for noncomplicated CD and postoperative endoscopic evaluation within 1 year in the Netherlands compared with Canada and France,^71^ may contribute to between-study heterogeneity and partly explain differences in recurrence rates in the biologics era.

In line with earlier observations, this study confirms a long-term decline in surgical recurrence rates from 1932 to 2020.^14^ Notably, the decline in surgical recurrence rates starts prior to the introduction of anti-TNF agents, followed by a stabilization of the trend thereafter. This observation was previously reported in population-based studies^5^^,^^59^^,^^69^ and suggests that long-term improvements in surgical recurrence rates are influenced by multiple factors rather than by advanced therapies alone. For instance, the widespread use of immunomodulators, later followed by anti-TNF agents,^72^ together with advancements in postoperative disease monitoring and medication strategies,^11^^,^^73^ have likely contributed to the reduction in recurrence rates. To adequately evaluate the impact of postoperative strategies on surgical recurrence rates, individual-participant data meta-analyses using data from high-quality registries could allow secular trends to be disentangled from study-specific effects. In particular, such studies could account for between-patient differences in terms of (prophylactic) medication use and could assess the potential impact of postoperative follow-up strategies on surgical recurrence rates. Evidence from previous studies suggests that surveillance colonoscopy at 6 months with treatment escalation is more effective in preventing CD recurrence than medical therapy without standardized endoscopic assessment.^73^ Moreover, prophylactic treatment in patients at high risk of recurrence, particularly with infliximab, is associated with lower rates of endoscopic recurrence.^74^ Therefore, current postoperative treatment strategies incorporating risk stratification and surveillance endoscopy, enabling timely initiation or optimization of therapy, are likely to improve recurrence outcomes.

After 2000, recurrence rates after ileocolic resection have largely plateaued rather than continue to decline. Several factors may explain this trend. First, the introduction of advanced therapies may have shifted the patient population undergoing ileocolic resection, with these patients now representing more medically refractory cases. This change could potentially contribute to the stabilization or even increase in recurrence rates after 2000. Furthermore, disease location influences the risk of recurrence, with ileocolonic (L3) CD being associated with a higher risk compared to terminal ileal (L1), as colonic involvement suggests a more extensive disease course and increased likelihood of postoperative disease progression.^57^^,^^75^ This study included both L1 and L3 subtypes, as these terms are often used interchangeably, but we cannot rule out the possibility that disease subtype influences recurrence rates. Additionally, heterogeneity between studies (including variations in patient characteristics, length of study inclusion periods, and postoperative treatment regimens) makes comparisons more challenging and increase the difficulty of detecting clear trends in recurrence rates.

Current guidelines incorporate several risk factors associated with POR, including active smoking, prior resections, a penetrating disease pattern, and perianal disease.^76^^,^^77^ In these guidelines, POR is defined broadly, comprising endoscopic, clinical, and surgical POR, and the identified risk factors are largely derived from earlier studies with inconsistent findings.^78^ To better understand risk factors for surgical recurrence in the biologic era, we analyzed the included studies. However, due to heterogeneity in risk factor reporting, a formal meta-analysis was feasible for only 2 variables: smoking and sex. Although smoking is a well-established risk factor for disease recurrence in the literature,^79^^,^^80^ we were unable to confirm a clear association with surgical recurrence. This may be due to the small number of available studies and sample sizes, resulting in wide 95% CIs, or it could reflect the potential impact of modern medical therapies on the relationship between smoking and surgical recurrence. Although none of the previously mentioned risk factors were significantly associated with surgical recurrence, they should not be dismissed as unimportant. The presence of these factors likely contributes to the variability in surgical recurrence rates and identifying them could improve our understanding of the factors influencing recurrence. However, high-quality, larger studies will be required to confirm these associations. In addition, Bak et al. reported a correlation between a higher modified Rutgeerts score and an increased risk of surgical recurrence, with a score of ≥ i2b being associated with surgical recurrence.^70^ This finding highlights the importance of close monitoring in this patient group and the potential effect of early postoperative monitoring and treatment on recurrence rates. Although some studies have examined the effect of postoperative (prophylactic) medication, the impact of biologic therapy on surgical recurrence rates could not be assessed in this systematic review due to its design.

This study has several limitations that should be acknowledged. To ensure the quality of the meta-analysis estimating the risk of surgical recurrence in the biologics era, only studies that reported 5- and 10-year cumulative recurrence rates with corresponding 95% CIs could be included. As a result, some studies were excluded, potentially introducing selection bias. To minimize this risk, we contacted authors for missing data or calculated CIs ourselves, resulting in usable data for 96% of the potentially eligible patients. Additionally, our analysis included various study designs, such as population-based, retrospective, and prospective cohort studies. While this enhances generalizability, it also introduced heterogeneity due to differences in patient selection, treatment strategies, and follow-up durations. Population-based studies provide real-world insights by including a broad spectrum of patients, whereas retrospective cohort studies often focus on more specific populations. This variation potentially contributes to the different cumulative 10-year surgical recurrence rates reported in several studies over the past 2 decades (Figure 2B, Table 1). Since the meta-analysis was based on aggregate rather than individual participant data, it was not possible to conduct stratified analyses based on patient-level characteristics (eg, disease severity and medical treatment), to explore whether these factors could explain between-study heterogeneity in the reported recurrence rates. Finally, as noted earlier, we have included both L1 and L3 disease, which may potentially affect recurrence rates.

This is the first review, to our knowledge, to focus exclusively on surgical recurrence after primary ICR in CD, offering a more accurate contemporary risk estimate for this specific patient group. The findings of this meta-analysis are highly relevant for both clinicians and patients. Updated surgical recurrence estimates in CD provide a more accurate understanding of long-term outcomes in an evolving treatment landscape and can help guide therapeutic decision-making.

## Conclusion

In conclusion, in the current biologics era, approximately 1 in 17 patients (5.7%) experiences surgical recurrence within 5 years after primary ICR, and 13% within 10 years. No clear risk factors for surgical recurrence were identified. Over the past decades, the risk of surgical recurrence following primary ICR for terminal ileitis in Crohns disease has declined, likely due to advances in treatment, including postoperative medical therapy and improved disease surveillance, leading to better long-term outcomes. However, since the introduction of biologic agents such as anti-TNF therapy, this risk appears to have stabilized.

## Supplementary Material

izag037_Supplementary_Data

## Data Availability

The data underlying this article are available in the article, in its online supplementary material, or in the studies cited in this article. Requests to obtain individual participant data of the primary studies should be directed to the corresponding authors of those respective studies.
