# Supplementary material for: Incidence and predictors of surgical recurrence after primary ileocolic resection in Crohn ileitis: a systematic review and meta-analysis
Source: Inflamm Bowel Dis. 2026 Apr 19;32(9):1815–32. doi: 10.1093/ibd/izag037 (PMC13533195; doi:10.1093/ibd/izag037)
Supplement: Supplementary file 1 [file izag037_supplementary_data.zip › Appendix 2. supplementary materials.docx]

# Appendix 2. Supplementary material

**Supplementary File 1.** Literature search

| Database searched 18-10-2019  **Total hits before deduplication:** 3369  **Total hits (unique):** 2698 | **Search string** |
| --- | --- |
| **Pubmed**  (N=1420) | ("Crohn Disease"[Mesh] OR crohn*[tiab]) AND ("Digestive System Surgical Procedures"[Mesh] OR reoperation*[tiab] OR resection*[tiab] OR surgical[tiab] OR surger*[tiab]) AND ("Recurrence"[Mesh] OR surgical recurren*[tiab])  NOT  ("Letter" [Publication Type] OR "Editorial" [Publication Type] OR "Comment" [Publication Type] OR letter[ti] OR editorial[ti] OR comment[ti])  NOT  (("Animals"[Mesh] OR "Animal Experimentation"[Mesh] OR "Models, Animal"[Mesh] OR animal experiment*[tiab] OR animal model*[tiab]) NOT "Humans"[Mesh]) |
| **Embase**  (N=1793) | Database(s): Embase Classic+Embase 1947 to 2019 October 17. Search Strategy: 1 \| exp Crohn disease/ or crohn*.ti,ab,kw. \| 102384; 2 \| recurrent disease/ and reoperation/ \| 5733; 3 \| ((reoperati* or resection* or surgical or surger*) adj6 (repeat* or recurrenc*)).ti,ab,kw. \| 66183; 4 \| 2 or 3 \| 70791; 5 \| 1 and 4 \| 1844; 6 \| (exp animal/ or exp animal experiment/ or exp animal model/ or (animal experiment* or animal model*).ti,ab,kw.) not human/ \| 5823781; 7 \| 5 not 6 \| 1824; 8 \| letter/ or editorial/ or (letter or editorial or comment).ti. \| 1745590; 9 \| 7 not 8 \| 1793 |
| **Cochrane Library**  (N=156) | Cochrane Library. Issue 10 of 12, October 2019  #1 (crohn*):ti,ab,kw 4757  #2 ((reoperati* or resection* or surgical or surger*) near/6 (repeat* or recurrenc*)):ti,ab,kw 5888  #3 #1 and #2 156 |

| Search update 15-11-2023  **Total hits before deduplication:** 992  **Total hits (unique):** 800 | **Search string** |
| --- | --- |
| **Pubmed**  (N=274) | ("Crohn Disease"[Mesh] OR crohn*[tiab]) AND ("Digestive System Surgical Procedures"[Mesh] OR reoperation*[tiab] OR resection*[tiab] OR surgical[tiab] OR surger*[tiab]) AND ("Recurrence"[Mesh] OR surgical recurren*[tiab])  NOT  ("Letter" [Publication Type] OR "Editorial" [Publication Type] OR "Comment" [Publication Type] OR letter[ti] OR editorial[ti] OR comment[ti])  NOT  (("Animals"[Mesh] OR "Animal Experimentation"[Mesh] OR "Models, Animal"[Mesh] OR animal experiment*[tiab] OR animal model*[tiab]) NOT "Humans"[Mesh])  AND ("2019/10/18"[Date - Publication]: "2023/11/15"[Date - Publication]) |
| **Embase**  (N=659) | Database(s): **Embase Classic+Embase** 1947 to 2023 November 13 Search Strategy: 1 \| exp Crohn disease/ or crohn*.ti,ab,kw. \| 130802; 2 \| recurrent disease/ and reoperation/ \| 7215; 3 \| ((reoperati* or resection* or surgical or surger*) adj6 (repeat* or recurrenc*)).ti,ab,kw. \| 86341; 4 \| 2 or 3 \| 92070; 5 \| 1 and 4 \| 2416; 6 \| (exp animal/ or exp animal experiment/ or exp animal model/ or (animal experiment* or animal model*).ti,ab,kw.) not human/ \| 6485189; 7 \| 5 not 6 \| 2390; 8 \| letter/ or editorial/ or (letter or editorial or comment).ti. \| 2067376; 9 \| 7 not 8 \| 2348; 10 \| limit 9 to yr="2019 -Current" \| 659 |
| **Cochrane Library**  (N=59) | [Cochrane Database of Systematic Reviews](https://www.cochranelibrary.com/): Issue 11 of 12, November 2023  [Cochrane Central Register of Controlled Trials](https://www.cochranelibrary.com/): Issue 10 of 12, October 2023  #1 (crohn*):ti,ab,kw 5855  #2 ((reoperati* or resection* or surgical or surger*) near/6 (repeat* or recurrenc*)):ti,ab,kw 8938  #3 #1 and #2 with Cochrane Library publication date Between Oct 2019 and Nov 2023 59 |

| Supplementary Table 1. characteristics of included studies reporting overall surgical recurrence rates | | | | | | | | | | | | | |
| --- | --- | --- | --- | --- | --- | --- | --- | --- | --- | --- | --- | --- | --- |
| Study, publication year | **Country, study design** | **Study span (years)** | **Sample size (primary ICR)** | **Follow-up duration after ICR (years)** | **Age at surgery (years)** | **Sex, (male, %)** | **Smokers (yes, %)** | **Disease behaviour** | **Disease location** | **Medication preoperative (primary ICR) (n, (%))** | **Medication postoperative following primary ICR (n, (%))** | **Overall risk surgical recurrence (n (%))** | **Clinical recurrence/ Endoscopic recurrence** |
| Gump^1^. 1960 | USA, retro SC cohort | 1932 - 1957 | 67 | Mean 6.7 ^a^ *(SD NR)* | NR | NR | NR | NR | NR | NR | NR | 10 (15) | NR |
| Lennard-Jones^2^, 1967 | UK, retro MC cohort | 1947-1965 | 71 | NR | Mean 36 (range 16-23) | 42 (59) | NR | NR | NR | NR | NR | 12 (17) | NR |
| Alexander-Williams^3^, 1972 | UK, retro SC cohort | 1950-unkown | 89 | Mean 14 (range 4-38) | NR | NR | NR | NR | NR | NR | NR | 46 (52) | NR |
| Young^4^, 1975 | UK, retro MC cohort | 1961-1970 | 140 | Range 1-27 *Mean or median NR* | NR | NR | NR | NR | NR | NR | NR | 51 (36) | NR |
| Homan^5^, 1978 | USA, retro SC cohort | 1932-1975 | 102 | NR | Mean 33 *(SD NR)* | NR | NR | NR | NR | CS: 26 (26) | NR | 25 (25) | NR |
| Higgens ^6^, 1980 | UK, retro SC cohort | 1944-1979 | 180 | NR | NR | NR | NR | NR | L1: 180 (100) | NR | NR | 72 (40) | NR |
| Lock^7^, 1981 | USA, retro SC cohort | 1955-1973 | 146 | Mean 12 ^a^  *(SD NR)* | Mean 32 ^b^ (range 7-72) | 178 ^b^ (49) | NR | NR | NR | NR | NR | 64 (44) |  |
| Whelan^8^, 1985 | USA, retro SC cohort | 1966-1969 | 225 | Mean 13 ^a^ *(SD NR)* | NR | 224 ^b^ (52) | NR | NR | NR | NR | NR | 119 (53) | NR |
| Adloff^9^, 1987 | France, retro SC cohort | 1972-1983 | 46 | Range 3-15  *Mean or median NR* | Mean 32 (range 13-61) | 26 (45) | NR | NR | L1: 15 (33)  L3: 31 (67) | NR | NR | 16 (35) | NR |
| Kangas^10^, 1987 | Finland, retro MC cohort | 1962-1985 | 41 | Median 8 (range <1- 23) | Median 35^b^ (range 15-70) | 35^b^ (48) | NR | NR | NR | NR | NR | 8 (20) | NR |
| Shivananda^11^, 1989 | NL, retro MC cohort | 1979-1983 | 68 | Mean 7 ^a^ *(SD NR)* | NR | 53^b^ (45) | NR | NR | L1: 35^b^ (30)  L3: 54^b^ (46) | NR | NR | 16 (24) | NR |
| Wettergren^12^, 1991 | Denmark, retro SC cohort | 1961-1987 | 48 | Median 10 (range 3-27) | Median 32 (range 12-65) | 17 (34) | NR | NR | NR | NR | NR | 9 (19) | NR |
| Andrews^13^, 1991 | UK, retro SC cohort | 1970-1988 | 110 | Mean 10 (range 7-11) | NR | NR | NR | NR | NR | CS: 23 (21) | NR | 33 (30) | NR |
| Nordgren^14^, 1994 | Sweden, pros SC cohort | 1968-1977 | 68 | Median 17^a^ (15-18) | Median 27^b^ (IQR 23-34) | NR | NR | NR | L1: 68 (100) | NR | NR | 31 (46) | NR |
| Sachar^15^, 1996 | USE, retro SC cohort | 1961-1984 | 71 | Median 9 *(IQR NR)* | Median 23 *(IQR NR)* | 35 (49) | NR | B2: 36 (51)  B3: 35 (49) | NR | NR | NR | 18 (25) | NR |
| Poggioli^16^, 1996 | Italy, pros SC cohort | 1978-1993 | 157 | Range 2-16 yr  *Mean or median NR* | Mean ^b^ 36 (±1) | 120^b^ (44) | NR | NR | NR | NR | NR | 10 (6.1) | NR |
| De Jong^17^, 1996 | NL, pros SC cohort | 1982-1987 | 50 | Mean 8 (Range 1-3) ^a^ | Median 30 ^b^ (range 14-70) | 30 (50) ^b^ | NR | NR | NR | NR | NR | 12 (24) | ER <1 year: 34 (68%) |
| Kim^18^, 1997 | USA, retro SC cohort | 1970-1993 | 181 | Median 14 (*IQR NR)* | Mean 33 (±1) | 79 (44) | NR | B3:50 (28) | NR | NR | NR | 45 (25) | NR |
| Moskovitz^19^, 1999 | Canada, retro SC cohort | NR | 92 | Mean 8 *(SD NR)* | Mean 31 (range 15-78) | 48 (52) | 16 (17) | NR | NR | NR | NR | 25 (27) | Sympt: 55% |
| Yamamoto^20^, 1999 | USA, retro MC cohort | 1975-1995 | 165 | Median 7 (range <1-23) | Median 29 (range 13-71) | 57 (37) | 77 (47) | B1/B2: 93 (56)  B3: 72 (44) | NR | NR | CS: 66 (40)  5-ASA: 30 (18) | 71 (43) | NR |
| Thaler^21^, 2005 | USA, pros SC cohort | 1992-2000 | 37 | Mean 4 (±2) | NR | 14 (38) | NR | NR | NR | NR | NR | 2 (5) | NR |
| Landsend^22^, 2006 | Norway, retro SC cohort | 1954-2002 | 32 | Median 27 ^a^  (range 20-49) | Median 24 (range 13-65) | 25 (47) ^b^ | NR | B1: 8 (15) ^b^ | L1: 20 (38) ^b^  L3: 24 (45) | NR | NR | 17 (53) | NR |
| Lowney^23^, 2006 | USA, retro SC cohort | 1987-2003 | LICR: 63 | Mean 5 *(SD NR)* | Median 35 (range 18-76) | 26 (41) | NR | NR | NR | CS: 29 (46)  Imm: 15 (24) | Chemoproph: 25 (39) | 6 (10) | NR |
|  |  |  | OICR:50 | Mean 7 *(SD NR)* | Median 37 (range 17-78) | 17 (34) | NR | NR | NR | CS: 25 (50)  Imm: 13 (26) | Chemoproph: 27 (54) | 12 (24) |  |
| Cullen^24^, 2007 | Ireland, retro SC cohort | 1980-2000 | 139 | Median 7 ^a^ (IQR 5 –11) | Median 29 (IQR 24-35) | 55 (40) | 68 (49) | B1: 88 (63)  B2: 18 (13)  B3: 33 (24) | NR | NR | NR | 49 (35) | Disease recurrence: 72 (52) |
| Unkart^25^, 2008 | USA, retro SC cohort | 1970-2007 | 176 | NR | Median 30 (range 12-75) | 70 (40) | 62 (35) | B1: 15 (8)  B2: 66 (38)  B3: 95 (54) | L1: 149 (85)  L3 27 (15) | NR | 5-ASA: 104 (59)  Imm: 69 (39)  Bio: 36 (20) | 53 (30) | NR |
| Stocchi^26^, 2008 | USA, pros SC cohort | 1994-1998 | 56 | Mean 11 (± 3) | Mean 36 *(SD NR)* | 24 (43) | 21 (38) | NR | NR | NR | CS: 12 (7)  AZA/MTX: 10 (18)  Bio: 9 (16) | 11 (20) | NR |
| Eshuis^27^, 2008 | NL, retro MC cohort | 1995-1998 | OICR: 44 | Median 9 (range 4-10) | NR | 10 (23) | 21 (48) | NR | NR | NR | Proph:  TP: 8 (18)  MTX: 1 (2)  5-ASA: 13 (30) | 10 (23) |  |
|  |  |  | LICR:27 | Median 9 (range 6-10) | NR | 7 (26) | 14 (52) | NR | NR | NR | Proph:  5-ASA: 12 (44) | 6 (22) |  |
| Alessandroni^28^, 2010 | Italy, case-control study | 1999-2004 | Total:200  OICR: 100 | Mean 5 *(SD NR)* | Median 35 (range 17-68) | 111 (56) | NR | 72 (36) | NR | NR | NR | 21 (11) | NR |
|  |  |  | LIRC: 100 | Mean 4 *(SD NR)* | Median 33 (range 17-66) |  |  |  |  |  |  |  |  |
| Vigo^29^, 2011 | Spain, retro SC cohort | 2000-2009 | 78 | Mean 7 (range 5-9) | Mean 38 (range 13-75) | 44 (57) | 18 (23) | NR | NR | B3: 45 (58) | NR | 12 (15) | NR |
| De Cruz^30^, 2012 | Australia, retro MC cohort | 1998-2008 | 97 | Median 4 (range 0-11) | Mean 33 (SD NR) | 65 (48)^b^ | NR | B1: 14 (10) ^b^  B2: 85 (63) ^b^  B3: 27 (20) ^b^ | L1:62 (46) ^b^  L3: 66 (49) ^b^ | CS: 103 (76) ^b^  MTX: 10 7) ^b^  TP: 52 (38) ^b^  Bio: 13 (10) ^b^ | Proph: 110 (81) ^b^  TP+MTX: 49 (45) ^b^ | 6 (6) | NR |
| Burke ^31^, 2013 | Ireland, retro SC cohort | 1981-2009 | 147 | Median 7 (IQR 2-13) | Mean 33 (±12) | 67 (46) | 62 (46) |  |  | CS: 106 (89)  TP: 58 (40)  Bio: 35 (244) | 38 (35), Med NS | 32 (22) | CR: 51% mean time to CR 5 year ± 5 |
| Rink^32^, 2014 | Switzerland, retro SC cohort | 1994-2001 | 115 | Median 9 (range 6-14) | Median 32 (ramge14-64) | 37 (31) | NR | NR | NR | 5-ASA: 72 (63)  CS: 71 (62)  AZA: 13 (11) | FU1 (5 yr post op):  5-ASA: 37 (35)  CS: 7 (7)  AZA: 11 (11)  Bio: 0 (0) | 14 (12) | NR |
| Riss^33^, 2014 | Austria, retro SC cohort | 1997-2006 | 116 | Mean 8 (±3) | Mean 33 (±12) | 66 (57) | 38 (33) | NR | NR | NR | Bio: 13 (11)  AZA/MTX: 65 (48) | 10 (8.6) | NR |
| Manser^34^, 2014 | Switzerland, pros MC cohort | 2006-2012 | 305 | Median 15 (range 0-25) | NR | 150 (49) | 133 (44) | B1:44 (14)  B2: 84 (28)  B3: 177 (58) | L1: 80 (26)  L3: 180 (59) | NS | NS | 80 (26) | NR |
| Toh^35^, 2018 | Australia, retro MC cohort | 1970-2009 | 213 | Mean ^a^ 13 (±9) | NR | NR | NR | NR | NR | NR | NR | 15 (7) | NR |
| Guo^36^, 2018 | China, retro SC cohort | 2006-2015 | 166 | Mean 4 (±2) | Mean 37 (±11) | 112 (67) | 22 (13) | B1: 8 (5)  B2: 64 (39) B3: 94 (57) | L1: 46 (28)  L3: 105 (63) | CS: 6 (4)  Bio: 13 (8)  AZA: 68 (41) | Proph:  AZA: 91 (54.8) | 45 (27) | CR: 33 (20)  ER: 42 (25) |
| Bolckmans ^39^, 2019 | UK, retro MC cohort | 2000-2012 | C1: 305  (one-stage) | Median  9 (IQR 6-12) | Median 35 (IQR 26-46) | 141 (46) | 60 (20) | B3: 85 (28) | L1: 164 (54)  L3: 133 (44) | NR | Proph:125/242 (54)  Bio: 41 (17)  TP: 60 (25)  MTX: 2 (<1) | 47 (15) | NR |
|  |  |  | C2: 77  (two-stage) | Median  8 (IQR 7-10) | Median 33 (IQR 24-45) | 45 (58) | 25 (32) | B3: 40 (52) | L1: 45 (58)  L3: 32 (42) | NR | Proph: 42/72 (58)  Biologics: 7 (11)  TP: 20 (25) | 11 (14) | NR |
| Coffey^38^, 2019 | Ireland, retro SC cohort | 2004-2010 | MS: 30 | Mean 6 (±4) | 38 (±14) | 14 (47) | 20 (67) | B1: 16 (53)  B2: 6 (20)  B3: 8 (27) | L1: 23 (77)  L3: 5 (17) | CS: 13 (43)  Imm: 11 (37)  Bio: 5 (17) | Proph:  TP: 4 (13)  Bio: 2 (7) | 12 (40) | NR |
|  |  | 2010-NR | EM: 34 | Mean 4 (±2) | 36 (±12) | 14 (41) | 20 (59) | B1: 8 (24)  B2: 14 (41)  B3: 12 (35) | L1: 26 (76)  L3: 6 (18) | CS: 12 (35)  Imm: 10 (29)  Bio: 15 (44) | Proph:  TP: 4 (12)  Bio 4 (12) | 1 (3) |  |
| Wasmann^37^, 2020 | NL, retro SC, cohort | 2002-2009 | 106 | Median 9 (IQR 6-11) | Mean 32 ± 13 | 36 (34) | 26 (25) | B3: 35 (33) | L1: 79 (74)  L3: 27 (26) | CS: 25 (24)  MTX: 45 (43)  Bio: 26 (25) | Proph:  Bio: 7 (7)  Imm: 33 (31) | 2 (2) | NR |
| Coletta^40^, 2019 | Italy, retro SC cohort | 2004-2016 | 118 | Median 5 (range <1-13) | NR | 64 (54) | 47 (40) | B1: 12 (10)  B2: 63 (53)  B3: 43 (36) | L1: 105 (89)  L3: 13 (11) | CS: 4 (3)  TP+Bio:71 (60) | 5-ASA: 40 (34)  CS: 37 (31)  TP+Bio: 38 (32) | 11 (9) | Medical relapse: 42% |
| Stevens^41^, 2020 | NL, retro MC cohort | 2008-2015 | 69 | Median 5 (IQR 3-8) | Mean 33 ± 14 | 24 (35) | 21 (30) | B1: 96 (100) | L1: 69 (100) | NR | Imm: 26 (38) | 0 (0) | NR |
| Ikeuchi^42^, 2021 | Japan, retro SC cohort | 1990-2015 | 169 | Median 13 (range 4-26) | Mean 30 (range 14-76) | 117 (69) | NR | B2: 88 (52)  B3: 73 (43) | L1: 95 (56)  L3: 67 (41) | NR | NR | 35 (21) | NR |
| Zemel^43^, 2021 | Israel, retro SC cohort | 2000-2015 | 202 | Median 3 (range 1-7) | Median 29 (range 24-39) | 106 (52) | 59 (29) | B2: 81 (40)  B3: 114 (56) | L1: 133 (66) | CS: 49 (24)  5-ASA: 59 (29)  TP: 69 (34)  Bio: 70 (35) | NR | 7 (3) | NR |
| King^44^, 2021 | UK, NHS Hospital statistics database | 2007-2016 | 12.230 | 6 (IQR 4-9) | 36 (IQR 26-50) | 5475 (44.8) | NR | NR | NR | NR | NR | 1367 (11) | NR |
| Qian^45^, 2022 | China, retro SC cohort | 2007-2019 | 404 | 6 (IQR 4-8) | NR | 279 (69) | 9 (2) | B1: 10 (3)  B2: 210 (52)  B3: 184 (46) | L1: 58 (14)  L3: 325 (80) | 5-ASA: 138 (34)  CS: 21 (5)  Imm: 78 (19)  Bio: 26 (6) | AZA: 308 (76)  Bio: 99 (35) | 12 (3) | NR |
| Carvello ^46^, 2023 | Italy, retro MC cohort | 2008-2018 | 262  NR: 117  R: 145 | 5 (IQR 2-7) | Mean 34 (±13) | 137 (52) | 80 (31) | B1: 70 (27)  B2: 94 (36)  B3: 98 (37) | L1: 144 (55)  L3: 105 (40) | CS: 177 (68)  Bio: 109 (42)  Monoclonal antibodies: 25 (10) | Proph: 43 (16), not further specified. | 11 (4) | ER: 55%  CR: 25% |
|  |  |  |  | 5 (IQR 3-7) | Mean 36 (±14) |  |  |  |  |  |  |  |  |
| Assaf^47^, 2023 | Israel, retro SC cohort | 2010-2021 | 189 | Median 6 (range 1-1.5*[sic]* | Mean 34 (range 15-78) | 104 (55) | 40 (21) | B2: 71 (38)  B3: 76 (40)  B2+B3: 42 (22) | L1: 126 (67)  L3: 61 (32) | CS: 26 (14)  Bio: 36 (19) | CS: 9 (5)  TP: 44 (23)  Bio 88 (47) | 8 (4) | ER: 53%  CR: 42% |
| Aratari^48^, 2024 | Italy, retro, MC cohort | 1980-2020 | 657 | Mean 7 (±3) | Median 37 (IQR 27–49) | 348 (53) | 284 (43) | B1/B2: 455 (69)  B3: 202 (31) | NR | 5-ASA/AB: 358 (55)  CS: 221 (34)  Imm: 96 (15)  Bio: 88 (14) | 5-ASA/AB: 420 (64) CS: 128 (20)  Imm: 163 (25)  Bio: 185 (28) | 84 (13) | NR |
| SC: single centre, MC: multi centre, retro: retrospective, pros: prospective, NR: Not reported, proph: prophylactic, fist: fistulizing disease, perf: perforated disease, non-perf: non perforated disease, Bio: biologicals, CS: corticosteroids, 5-ASA: mesalazine, AB: Antibiotics, TP: thiopurines, Imm: immunosuppressive, B1: inflammatory, B2: fibrostenotic, B3: penetrating, L1: ileal, L2: colonic, L3: ileocolonic, LICR: laparoscopic ileocolic resection, OICR: Open ileocolic resection. EM: extended mesenteric excision, MS: Mesenteric sparing Excision. Non-R: non recurrent; R: recurrent. | | | | | | | | | | | | | |

^a^ Follow-up for the entire cohort and not specifically for those patients who underwent a primary ileocecal resection

^b^ Variable for the entire cohort and not specifically for those patients who underwent a primary ileocecal resection

| **Supplementary Table 2.** Risk of bias assessment | | | | | | | | | | | | |
| --- | --- | --- | --- | --- | --- | --- | --- | --- | --- | --- | --- | --- |
|  | **Newcastle-Ottawa Scale – cohort** | | | | | | | | | | | |
|  | **Selection** | | | | **Outcome** | | | |  | **Total score** | | **Overall** |
|  | **RC** | **S** | **A** | **D** |  | **AO** | **FU** | **AC** |  |  |  | |
| Gump et al. (1960) | * | * | * | * |  | * | * | * |  | 7 out of 7 | Good | |
| Lennard-Jones et al. (1967) | * | * | * | * |  | * | - | * |  | 6 out of 7 | Good | |
| Alexander-Williams et al. (1972) | * | * | * | * |  | * | * | * |  | 7 out of 7 | Good | |
| Young et al. (1975) | * | * | * | * |  | * | - | * |  | 6 out of 7 | Good | |
| Homan et al. (1978) | * | * | * | * |  | * | - | * |  | 6 out of 7 | Good | |
| Higgens et al. (1980) | * | * | * | * |  | * | - | * |  | 6 out of 7 | Good | |
| Lock et al. (1981) | * | * | * | * |  | * | * | * |  | 7 out of 7 | Good | |
| Whelan et al. (1985) | * | * | * | * |  | * | * | * |  | 8 out of 7 | Good | |
| Adloff et al. (1987) | * | * | * | * |  | * | - | - |  | 5 out of 7 | Poor | |
| Kangas et al. (1987) | * | * | * | * |  | * | * | - |  | 6 out of 7 | Good | |
| Shivananda et al. (1989) | * | * | * | * |  | * | * | - |  | 6 out of 7 | Good | |
| Wettergren et al. (1991) | * | * | * | * |  | * | * | - |  | 6 out of 7 | Good | |
| Andrews et al. (1991) | * | * | * | * |  | * | * | * |  | 7 out of 7 | Good | |
| Nordgren et al. (1994) | * | * | * | * |  | * | * | * |  | 7 out of 7 | Good | |
| Sachar et al. (1996) | - | - | * | * |  | * | * | * |  | 5 out of 7 | Fair | |
| Poggioli et al. (1996) | * | * | * | * |  | * | - | * |  | 6 out of 7 | Good | |
| De Jong et al. (1996) | - | * | * | * |  | * | * | - |  | 6 out of 7 | Good | |
| Kim et al. (1997) | * | * | * | * |  | * | * | - |  | 6 out of 7 | Good | |
| Moskovitz et al. (1999) | * | * | * | * |  | * | * | - |  | 7 out of 7 | Good | |
| Yamamoto et al. (1999) | - | * | * | * |  | * | * | * |  | 7 out of 7 | Good | |
| Thaler et al. (2005) | * | * | * | * |  | * | - | - |  | 5 out of 7 | Poor | |
| Landsend et al. (2006) | * | * | * | * |  | * | * | - |  | 6 out of 7 | Good | |
| Lowney et al. (2006) | - | * | * | * |  | * | * | - |  | 5 out of 7 | Good | |
| Cullen et al. (2007) | - | * | * | * |  | * | * | - |  | 5 out of 7 | Good | |
| Unkart et al. (2008) | * | * | * | * |  | * | - | * |  | 6 out of 7 | Good | |
| Stocchi et al. (2008) | - | * | * | * |  | * | * | * |  | 6 out of 7 | Good | |
| Eshuis et al. (2008) | * | * | * | * |  | * | * | * |  | 7 out of 7 | Good | |
| Alessandroni et al. (2010) | * | * | * | * |  | * | * | - |  | 6 out of 7 | Good | |
| Vigo et al. (2011) | * | * | * | * |  | * | * | * |  | 7 out of 7 | Good | |
| De Cruz et al. (2012) | * | * | * | * |  | * | - | - |  | 5 out of 7 | Poor | |
| Burke et al. (2013) | - | * | * | * |  | * | * | - |  | 5 out of 7 | Good | |
| Rink et al. (2014) | - | * | * | * |  | * | * | * |  | 6 out of 7 | Good | |
| Riss et al. (2014) | * | * | * | * |  | * | * | * |  | 7 out of 7 | Good | |
| Manser et al. (2014) | * | * | * | - |  | * | * | - |  | 5 out of 7 | Good | |
| Li et al. (2015) | * | * | * | * |  | * | * | - |  | 6 out of 7 | Good | |
| De Buck van Overstraeten et al. 2017 | * | * | * | * |  | * | * | * |  | 7 out of 7 | Good | |
| Toh et al. (2018) | * | * | * | * |  | * | * | * |  | 7 out of 7 | Good | |
| Guo et al. (2018) | * | * | * | * |  | * | - | - |  | 5 out of 7 | Poor | |
| Bolckmans et al. (2019) | * | * | * | * |  | * | * | - |  | 6 out of 7 | Good | |
| Coffey et al. (2019) | - | * | * | * |  | * | * | - |  | 5 out of 7 | Good | |
| Wasmann et al. (2020) | - | * | * | * |  | * | * | * |  | 6 out of 7 | Good | |
| Coletta et al. (2019) | * | * | * | - |  | * | * | - |  | 5 out of 7 | Good | |
| Stevens et al. (2020) | - | * | * | * |  | * | * | - |  | 5 out of 7 | Good | |
| Kalman et al. (2020) | * | * | * | - |  | * | * | - |  | 5 out of 7 | Good | |
| Ikeuchi et al. (2021) | * | * | * | * |  | * | * | - |  | 6 out of 7 | Good | |
| Beelen et al. (2021) | * | * | * | - |  | * | * | * |  | 6 out of 9 | Good | |
| Zemel et al. (2021) | * | * | * | - |  | * | - | * |  | 5 out of 7 | Good | |
| King et al. (2021) | * | * | * | - |  | * | * | - |  | 5 out of 7 | Good | |
| Quian et al. (2022) | * | * | * | * |  | * | * | * |  | 7 out of 7 | Good | |
| Mineccia et al. (2022) | * | * | * | * |  | * | * | - |  | 6 out of 7 | Good | |
| Carvello et al. (2023) | * | * | * | * |  | * | * | * |  | 7 out of 7 | Good | |
| Bislenghi et al. (2023) | - | * | * | * |  | * | * | - |  | 5 out of 7 | Good | |
| Assaf et al. (2023) | * | * | * | * |  | * | * | - |  | 6 out of 7 | Good | |
| Bak et al. (2023) | * | * | * | * |  | * | * | - |  | 6 out of 7 | Good | |
| Aratari et al. (2024) | * | * | * | * |  | * | * | - |  | 6 out of 7 | Good | |

Overview of the risk of bias assessment

Newcastle-Ottawa Scale (NOS) was used to assess the risk of bias in the included observational cohort and case-control studies. The thresholds for converting the NOS were as followed:

| **Good quality** | 3 or 4 stars in selection domain AND 2 or 3 stars in outcome/exposure domain |
| --- | --- |
| **Fair quality** | 2 stars in selection domain AND 2 or 3 stars in outcome/exposure domain |
| **Poor quality** | 0 or 1 star in selection domain OR 0 or 1 stars in outcome/exposure domain |

**Supplementary Figure 1. Sensitivity analysis excluding King et al.**

**Supplementary Figure 2. Sensitivity analysis excluding studies rated as ‘fair’ or ‘poor’ in the Newcastle-Ottawa scale.**

**References**

1.	Gump F, Lepore MJ. Prognosis in acute and chronic regional enteritis. *Gastroenterology* 1960;**39**:694-701.

2.	Lennard-Jones JE, Stalder GA. Prognosis after resection of chronic regional ileitis. *Gut* 1967;**8**:332-6.

3.	Alexander-Williams J, Fielding JF, Cooke WT. A comparison of results of excision and bypass for ileal crohn's disease. *Gut* 1972;**13**:973-5.

4.	Young S, Smith IS, O'Connor J, Bell JR, Gillespie G. Results of surgery for crohn's disease in the glasgow region, 1961-70. *Br J Surg* 1975;**62**:528-34.

5.	Homan WP, Dineen P. Comparison of the results of resection, bypass, and bypass with exclusion for ileocecal crohn's disease. *Ann Surg* 1978;**187**:530-5.

6.	Higgens CS, Allan RN. Crohn's disease of the distal ileum. *Gut* 1980;**21**:933-40.

7.	Lock MR, Farmer RG, Fazio VW*, et al.* Recurrence and reoperation for crohn's disease: The role of disease location in prognosis. *N Engl J Med* 1981;**304**:1586-8.

8.	Whelan G, Farmer RG, Fazio VW, Goormastic M. Recurrence after surgery in crohn's disease. Relationship to location of disease (clinical pattern) and surgical indication. *Gastroenterology* 1985;**88**:1826-33.

9.	Adloff M, Arnaud JP, Ollier JC. Does the histologic appearance at the margin of resection affect the postoperative recurrence rate in crohn's disease? *Am Surg* 1987;**53**:543-6.

10.	Kangas E, Matikainen M. Resection for the treatment of crohn's disease--a thirteen years experience in the tampere region. *Ann Chir Gynaecol* 1987;**76**:204-8.

11.	Shivananda S, Hordijk ML, Pena AS, Mayberry JF. Crohn's disease: Risk of recurrence and reoperation in a defined population. *Gut* 1989;**30**:990-5.

12.	Wettergren A, Christiansen J. Risk of recurrence and reoperation after resection for ileocolic crohn's disease. *Scand J Gastroenterol* 1991;**26**:1319-22.

13.	Andrews HA, Keighley MR, Alexander-Williams J, Allan RN. Strategy for management of distal ileal crohn's disease. *Br J Surg* 1991;**78**:679-82.

14.	Nordgren SR, Fasth SB, Oresland TO, Hultén LA. Long-term follow-up in crohn's disease. Mortality, morbidity, and functional status. *Scand J Gastroenterol* 1994;**29**:1122-8.

15.	Sachar DB, Subramani K, Mauer K*, et al.* Patterns of postoperative recurrence in fistulizing and stenotic crohn's disease. A retrospective cohort study of 71 patients. *J Clin Gastroenterol* 1996;**22**:114-6.

16.	Poggioli G, Laureti S, Selleri S*, et al.* Factors affecting recurrence in crohn's disease. Results of a prospective audit. *Int J Colorectal Dis* 1996;**11**:294-8.

17.	de Jong E, van Dullemen HM, Slors JF*, et al.* Correlation between early recurrence and reoperation after ileocolonic resection in crohn's disease: A prospective study. *J Am Coll Surg* 1996;**182**:503-8.

18.	Kim NK, Senagore AJ, Luchtefeld MA*, et al.* Long-term outcome after ileocecal resection for crohn's disease. *Am Surg* 1997;**63**:627-33.

19.	Moskovitz D, McLeod RS, Greenberg GR, Cohen Z. Operative and environmental risk factors for recurrence of crohn's disease. *Int J Colorectal Dis* 1999;**14**:224-6.

20.	Yamamoto T, Allan RN, Keighley MR. Perforating ileocecal crohn's disease does not carry a high risk of recurrence but usually re-presents as perforating disease. *Dis Colon Rectum* 1999;**42**:519-24.

21.	Thaler K, Dinnewitzer A, Oberwalder M*, et al.* Assessment of long-term quality of life after laparoscopic and open surgery for crohn's disease. *Colorectal Dis* 2005;**7**:375-81.

22.	Landsend E, Johnson E, Johannessen HO, Carlsen E. Long-term outcome after intestinal resection for crohn's disease. *Scand J Gastroenterol* 2006;**41**:1204-8.

23.	Lowney JK, Dietz DW, Birnbaum EH*, et al.* Is there any difference in recurrence rates in laparoscopic ileocolic resection for crohn's disease compared with conventional surgery? A long-term, follow-up study. *Dis Colon Rectum* 2006;**49**:58-63.

24.	Cullen G, O'Toole A, Keegan D*, et al.* Long-term clinical results of ileocecal resection for crohn's disease. *Inflamm Bowel Dis* 2007;**13**:1369-73.

25.	Unkart JT, Anderson L, Li E*, et al.* Risk factors for surgical recurrence after ileocolic resection of crohn's disease. *Dis Colon Rectum* 2008;**51**:1211-6.

26.	Stocchi L, Milsom JW, Fazio VW. Long-term outcomes of laparoscopic versus open ileocolic resection for crohn's disease: Follow-up of a prospective randomized trial. *Surgery* 2008;**144**:622-7; discussion 7-8.

27.	Eshuis EJ, Polle SW, Slors JF*, et al.* Long-term surgical recurrence, morbidity, quality of life, and body image of laparoscopic-assisted vs. Open ileocolic resection for crohn's disease: A comparative study. *Dis Colon Rectum* 2008;**51**:858-67.

28.	Alessandroni L, Bertolini R, Campanelli A*, et al.* Video-assisted versus open ileocolic resection in primary crohn's disease: A comparative case-matched study. *Updates Surg* 2010;**62**:35-40.

29.	Vigo MS, Grobas JP, Díaz MB*, et al.* [factors affecting the postoperative recurrence of crohn's disease. New controversies with one centre's experience]. *Cir Esp* 2011;**89**:290-9.

30.	De Cruz P, Bernardi MP, Kamm MA*, et al.* Postoperative recurrence of crohn's disease: Impact of endoscopic monitoring and treatment step-up. *Colorectal Dis* 2013;**15**:187-97.

31.	Burke JP, O'Connell RM, Lennon G*, et al.* The influence of ctgf single-nucleotide polymorphisms on outcomes in crohn's disease. *Ann Surg* 2013;**258**:767-73; discussion 73-4.

32.	Rink AD, Fischer IR, Vestweber B, Vestweber KH. Long-term outcome of laparoscopic ileocecal resection for crohn's disease before the era of biologics. *Int J Colorectal Dis* 2014;**29**:127-32.

33.	Riss S, Schuster I, Papay P*, et al.* Surgical recurrence after primary ileocolic resection for crohn's disease. *Tech Coloproctol* 2014;**18**:365-71.

34.	Manser CN, Frei P, Grandinetti T*, et al.* Risk factors for repetitive ileocolic resection in patients with crohn's disease: Results of an observational cohort study. *Inflamm Bowel Dis* 2014;**20**:1548-54.

35.	Toh JWT, Wang N, Young CJ*, et al.* Major abdominal and perianal surgery in crohn's disease: Long-term follow-up of australian patients with crohn's disease. *Dis Colon Rectum* 2018;**61**:67-76.

36.	Guo Z, Cai X, Liu R*, et al.* Immediate prophylactic vs endoscopic or symptomatic-driven azathioprine treatment to prevent surgical recurrence after intestinal resection for crohn's disease. *Colorectal Dis* 2018;**20**:O267-o76.

37.	Wasmann K, van Amesfoort J, van Montfoort ML*, et al.* The predictive value of inflammation at ileocecal resection margins for postoperative crohn's recurrence: A cohort study. *Inflamm Bowel Dis* 2020;**26**:1691-9.

38.	Coffey CJ, Kiernan MG, Sahebally SM*, et al.* Inclusion of the mesentery in ileocolic resection for crohn's disease is associated with reduced surgical recurrence. *J Crohns Colitis* 2018;**12**:1139-50.

39.	Bolckmans R, Singh S, Ratnatunga K*, et al.* Temporary faecal diversion in ileocolic resection for crohn's disease: Is there an impact on long-term surgical recurrence? *Colorectal Dis* 2020;**22**:430-8.

40.	Coletta M, Zefelippo A, Mazza S*, et al.* Previous colonic resection is a risk factor for surgical relapse in crohn's disease. *Dig Liver Dis* 2019;**51**:206-11.

41.	Stevens TW, Haasnoot ML, D'Haens GR*, et al.* Laparoscopic ileocaecal resection versus infliximab for terminal ileitis in crohn's disease: Retrospective long-term follow-up of the lir!C trial. *Lancet Gastroenterol Hepatol* 2020;**5**:900-7.

42.	Ikeuchi H, Uchino M, Bando T*, et al.* Localization of recurrent lesions following ileocolic resection for crohn's disease. *BMC Surg* 2021;**21**:145.

43.	Zemel M, Solo E, Tulchinsky H. Does microscopic involvement of the surgical margins after ileocecectomy in crohn's patients predict early recurrence? *Int J Colorectal Dis* 2021;**36**:2427-35.

44.	King D, Coupland B, Dosanjh A*, et al.* The risk of later surgery at the anastomotic site following right hemicolectomy for crohn's disease in a national cohort of 12 230 patients. *Aliment Pharmacol Ther* 2021;**53**:114-27.

45.	Qian W, Gao L, Huang L*, et al.* The prevalence of concomitant skip small bowel lesions in crohn's disease and their effects on reoperation in patients undergoing ileocolic resection. *J Gastrointest Surg* 2022;**26**:2330-41.

46.	Carvello M, D'Hoore A, Maroli A*, et al.* Postoperative complications are associated with an early and increased rate of disease recurrence after surgery for crohn's disease. *Dis Colon Rectum* 2023;**66**:691-9.

47.	Assaf D, Hazzan D, Laks S, Segev L. Long-term outcomes following ileocolic resection for crohn's disease: Does earlier elective resection affect disease recurrence rates? *ANZ J Surg* 2023;**93**:2910-20.

48.	Aratari A, Scribano ML, Pugliese D*, et al.* Crohn's disease after surgery: Changes in post-operative management strategies over time and their impact on long-term re-operation rate-a retrospective multicentre real-world study. *Aliment Pharmacol Ther* 2024;**59**:1579-88.
